# Disrupted uromodulin trafficking is rescued by targeting TMED cargo receptors

**DOI:** 10.1172/JCI180347

**Published:** 2024-12-16

**Authors:** Silvana Bazua-Valenti, Matthew R. Brown, Jason Zavras, Magdalena Riedl Khursigara, Elizabeth Grinkevich, Eriene-Heidi Sidhom, Keith H. Keller, Matthew Racette, Moran Dvela-Levitt, Catarina Quintanova, Hasan Demirci, Sebastian Sewerin, Alissa C. Goss, John Lin, Hyery Yoo, Alvaro S. Vaca Jacome, Malvina Papanastasiou, Namrata Udeshi, Steven A. Carr, Nina Himmerkus, Markus Bleich, Kerim Mutig, Sebastian Bachmann, Jan Halbritter, Stanislav Kmoch, Martina Živná, Kendrah Kidd, Anthony J. Bleyer, Astrid Weins, Seth L. Alper, Jillian L. Shaw, Maria Kost-Alimova, Juan Lorenzo B. Pablo, Anna Greka

**Affiliations:** 1 The Broad Institute of Massachusetts Institute of Technology (MIT) and Harvard, Cambridge, Massachusetts, USA.; 2 Department of Medicine, Brigham and Women’s Hospital and Harvard Medical School, Boston, Massachusetts, USA.; 3 Departamento de Nefrología y Metabolismo Mineral, Instituto Nacional de Ciencias Médicas y Nutrición Salvador Zubirán, Ciudad de México, México.; 4 Department of Pathology, Brigham and Women’s Hospital and Harvard Medical School, Boston, Massachusetts, USA.; 5 The Mina and Everard Goodman Faculty of Life Sciences, Bar-Ilan University, Ramat-Gan, Israel.; 6 Institute of Physiology, Christian - Albrechts - Universität, Kiel, Germany.; 7 Institute of Translational Physiology and; 8 Department of Anatomy, Charité - Universitätsmedizin, Berlin, Germany.; 9 Proteomics Platform, The Broad Institute of MIT and Harvard, Cambridge, Massachusetts, USA.; 10 Department of Nephrology and Medical Intensive Care, Charité - Universitätsmedizin, Berlin, Corporate Member of Freie Universität Berlin and Humboldt-Universität zu Berlin, Berlin, Germany.; 11 Research Unit for Rare Diseases, Department of Pediatrics and Adolescent Medicine, First Faculty of Medicine, Charles University, Prague, Czech Republic.; 12 Section on Nephrology, Wake Forest School of Medicine, Medical Center Blvd., Winston-Salem, North Carolina, USA.; 13 Division of Nephrology, Beth Israel Deaconess Medical Center and Department of Medicine, Harvard Medical School, Boston, Massachusetts, USA.

**Keywords:** Nephrology, Genetic diseases, Protein misfolding, Protein traffic

## Abstract

The trafficking dynamics of uromodulin (UMOD), the most abundant protein in human urine, play a critical role in the pathogenesis of kidney disease. Monoallelic mutations in the *UMOD* gene cause autosomal dominant tubulointerstitial kidney disease (ADTKD-*UMOD*), an incurable genetic disorder that leads to kidney failure. The disease is caused by the intracellular entrapment of mutant UMOD in kidney epithelial cells, but the precise mechanisms mediating disrupted UMOD trafficking remain elusive. Here, we report that transmembrane Emp24 protein transport domain–containing (TMED) cargo receptors TMED2, TMED9, and TMED10 bind UMOD and regulate its trafficking along the secretory pathway. Pharmacological targeting of TMEDs in cells, in human kidney organoids derived from patients with ADTKD-*UMOD*, and in mutant-*UMOD*-knockin mice reduced intracellular accumulation of mutant UMOD and restored trafficking and localization of UMOD to the apical plasma membrane. In vivo, the TMED-targeted small molecule also mitigated ER stress and markers of kidney damage and fibrosis. Our work reveals TMED-targeting small molecules as a promising therapeutic strategy for kidney proteinopathies.

## Abstract 2

In this original research article, we elucidate the pivotal role that Transmembrane Emp24 Protein Transport Domain Containing (TMED) cargo-receptors—specifically, TMED2, TMED9, and TMED10—play in the pathogenesis of a kidney disease associated with uromodulin (UMOD). Our findings demonstrate that TMEDs interact with UMOD, subsequently regulating its trafficking along the secretory pathway within kidney epithelial cells. Through *in vitro*, *ex vivo* and *in vivo* experiments, we determined that pharmacologically targeting these TMED proteins can significantly ameliorate the toxic accumulation of mutant misfolded UMOD. Our therapeutic interventions also restored the normal trafficking and localization of UMOD to the apical plasma membrane. These results not only underscore the crucial involvement of TMED cargo-receptors in the molecular mechanisms underlying UMOD-associated kidney pathologies but also highlight pharmacological targeting of these receptors as a promising therapeutic strategy for addressing kidney disease triggered by aberrant UMOD trafficking.

## Graphical abstract

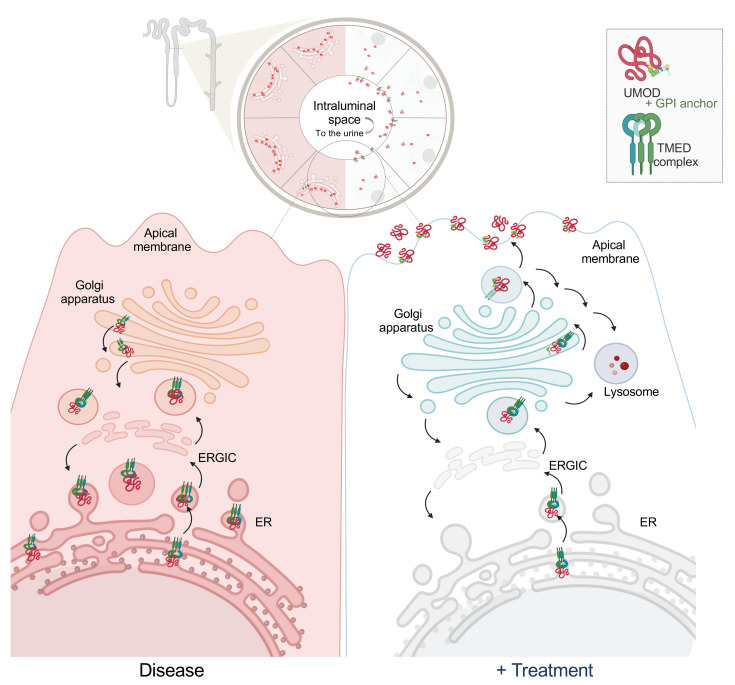

## Introduction

Uromodulin (UMOD; also known as Tamm-Horsfall protein) is the most abundant protein secreted in the urine of healthy individuals. UMOD is expressed exclusively in the epithelial cells of 2 specific segments of the kidney tubule called the thick ascending limb (TAL) of the loop of Henle and the early distal convoluted tubule (DCT1). In these cells, UMOD critically regulates the expression of channels and co-transporters essential to TAL physiology (1–4).

UMOD is a GPI-anchored protein composed of 4 epidermal growth factor–like (EGF-like) domains, 1 cysteine-rich domain (D8C), and 1 polymerization-promoting zona pellucida (ZP) domain divided into 2 segments (5). Newly synthesized UMOD traffics from the endoplasmic reticulum (ER) to the Golgi via COPII vesicles/tubules. UMOD is then trafficked to the plasma membrane where it is cleaved by the protease hepsin, leading to luminal secretion and urinary excretion of cleaved UMOD (6, 7). During this process, UMOD undergoes glycosylation, such that glycans ultimately contribute approximately 32% of its molecular mass (3, 8). The concentration of urinary UMOD is a promising prognostic or diagnostic biomarker to monitor physiologic and pathophysiologic states of the kidney (9). Lower urinary UMOD levels have been linked to acute kidney injury, tubulointerstitial damage, and progression of kidney disease, whereas elevated levels correlate with healthy kidney function (9, 10). In sum, dissecting the regulation of UMOD trafficking, glycosylation, and urinary excretion is crucial to understanding UMOD’s role in health and disease.

UMOD mutations cause monoallelic autosomal dominant tubulointerstitial kidney disease (ADTKD-*UMOD*, OMIM #16200), a non-proteinuric disorder with decreased fractional excretion of urate, early-onset gout, and a variable onset and gradual decline in kidney function leading to failure at approximately 54 years (11, 12). Of the 135 UMOD mutations reported to date, more than half alter a cysteine residue to impair protein folding. The misfolded mutant proteins are trapped in the early secretory pathway (13, 14). Mutant UMOD’s entrapment and accumulation activate the unfolded protein response (UPR) and ER-stress pathways (15–17). Previous studies have suggested parallel entrapment of wild-type UMOD (UMOD^WT^), preventing its normal trafficking and interfering with its physiological role, thereby contributing to the pathogenicity of UMOD mutations (18, 19). However, the molecular machinery and interacting proteins that control both mutant-UMOD and UMOD^WT^ trafficking remain elusive.

We recently showed that a frameshifted mutant mucin 1 protein, the cause of ADTKD-*MUC1* (phenotypically identical to ADTKD-*UMOD*), is trapped in transmembrane Emp24 protein transport domain–containing 9 (TMED9) cargo receptor–containing vesicles. This entrapment is released after treatment with the TMED-targeted small molecule BRD4780 (20). Furthermore, the trafficking of some GPI-anchored proteins (such as mutant prion protein) is facilitated by the TMED family of cargo receptors (21–24). We therefore hypothesized that TMEDs may play a role in the trafficking and regulation of UMOD. To test this hypothesis, we investigated the molecular mechanisms responsible for mutant UMOD handling in the secretory pathway in vitro and in vivo. We found that TMED2, TMED9, and TMED10 interact with UMOD and regulate its trafficking. A TMED-targeted small molecule promoted removal of intracellularly trapped mutant UMOD and restored plasma membrane localization of UMOD in cells and in human kidney organoids. Targeting these TMEDs in vivo led to an improvement in markers of kidney damage in a mouse model of ADTKD-*UMOD*.

## Results

### TMED cargo receptors bind UMOD.

To better understand the mechanisms that underlie intracellular entrapment of mutant UMOD, we sought to define the interactome of human UMOD mutant Cys126Arg (UMOD^C126R^) compared to UMOD^WT^. The Cys126Arg mutation is associated with a severe ADTKD-*UMOD* kidney disease phenotype (25, 26). As with many UMOD cysteine mutations, Cys126Arg is thought to disrupt native disulfide bond formation, promoting UMOD misfolding and ER retention, as evidenced by its strong colocalization with calnexin (25, 27) (Supplemental Figure 1A; supplemental material available online with this article; https://doi.org/10.1172/JCI180347DS1). Using immunoprecipitation–mass spectrometry (IP-MS), we found a distinct set of binding partners for UMOD^WT^ and UMOD^C126R^ (Supplemental Figure 1B and Supplemental Figure 2). Pathway analysis of the UMOD^WT^ interactome revealed terms predominantly related to organelle organization and membrane trafficking pathways (Supplemental Figure 2, A and D). These findings are consistent with UMOD’s established role in cellular homeostasis of TAL epithelial cells. Conversely, the interaction profile of UMOD^C126R^ diverged significantly, demonstrating association with extracellular matrix organization (Supplemental Figure 2, B and E).

**Figure 1:**
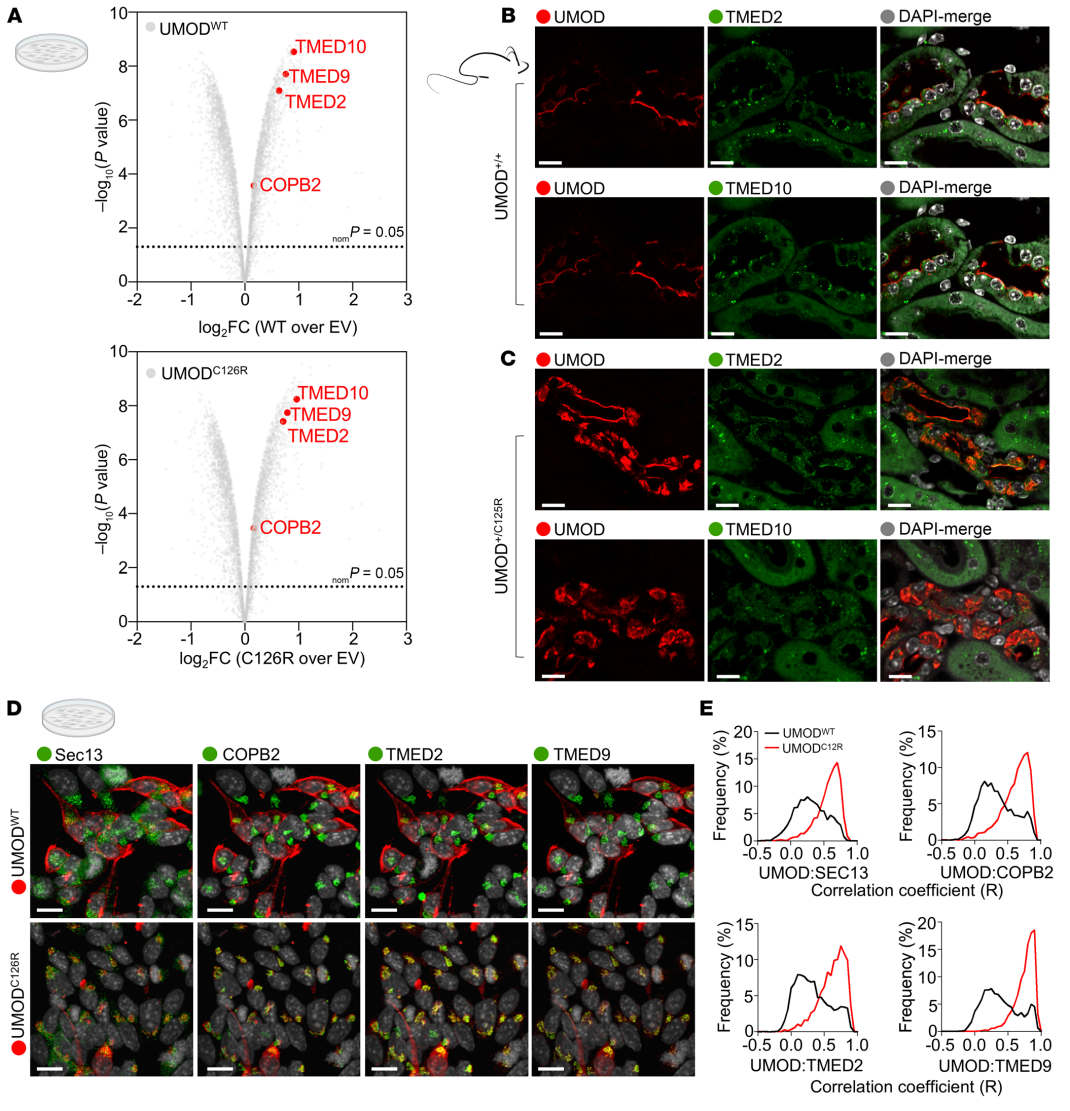
UMOD localizes and interacts with the TMED family of cargo receptors. (**A**) Volcano plots depicting proteomes of FLAG-tagged WT UMOD (UMOD^WT^) or mutant misfolded UMOD (UMOD^C126R^) in HEK293T cells. Significant interactions with TMED2, TMED9, TMED10, and COPB2 (COPI protein) are indicated (red dots). Two-sample moderate *t* test (2 tailed). *n* = 3 technical replicates. EV, empty vector; FC, fold change. (**B** and **C**) Immunofluorescence images of kidney sections from 10-month-old WT (UMOD^+/+^) or heterozygous knockin (UMOD^+/C125R^) mice stained for UMOD (red), for TMED2 or TMED10 (green), and with DAPI (gray). Scale bars: 10 μm. TMED2 and TMED10 images were obtained from consecutive sections to facilitate comparison. (**D**) Representative immunofluorescence of AtT-20 cells stably transfected with UMOD^WT^ or UMOD^C126R^, following fixation and the 4i protocol. Scale bars: 10 μm. (**E**) Histograms representing single-cell correlation coefficients between UMOD and selected secretory pathway markers.

TMED2, TMED9, and TMED10 emerged among the most highly enriched proteins in both UMOD^WT^ and UMOD^C126R^ immunoprecipitates (Figure 1A). Coimmunoprecipitation (co-IP) experiments with candidate TMEDs coexpressed with UMOD in HEK293 cells confirmed the results from IP-MS experiments (Supplemental Figure 1C). To further validate specific UMOD binding to TMEDs, we generated a TMED9 deletion mutant (TMED9ΔGOLD) lacking the Golgi dynamics (GOLD) domain thought to be important for binding to cargo proteins (28, 29). Immunoprecipitation of Myc-tagged TMED9 revealed its interaction with both UMOD^WT^ and UMOD^C125R^; however, this binding was eliminated in the absence of the GOLD domain (TMED9ΔGOLD; Supplemental Figure 1D).

Analysis of an independently published dataset confirmed the abundant expression of TMED2, TMED9, and TMED10 in TAL epithelial cells in vivo (30) (Supplemental Figure 3A). To corroborate the UMOD-TMED interaction in vivo, we performed co-IP assays with whole-kidney lysates from WT (UMOD^+/+^) mice and mice homozygous for the Cys125Arg mutation, which is homologous to the human Cys126Arg mutation (UMOD^C125R/C125R^ mice). UMOD^+/+^ mice should express only properly folded UMOD, while UMOD^C125R/C125R^ mice should express only misfolded mutant UMOD. These co-IP experiments revealed interaction of TMED9 and TMED2 with both WT and mutant UMOD (Supplemental Figure 3B).

The newly validated interaction between UMOD and TMED cargo receptors led us to investigate the localization of TMEDs in TAL epithelial cells where UMOD is prominently expressed on the apical membrane (Figure 1B). We found that, like UMOD, TMED2 and TMED10 were expressed in TAL cells (Figure 1B). TMED2 was distributed throughout the cytoplasm and in a perinuclear pattern, whereas abundant TMED10 was localized mainly in subapical puncta in the same cells (Figure 1B). We hypothesized that ADTKD-causing mutations in UMOD lead to intracellular localization in the same compartments where TMEDs reside. Consistent with this hypothesis, TMED2 and TMED10 colocalized with intracellular UMOD in TAL cells of UMOD^+/C125R^ heterozygous mice (Figure 1C).

To examine the subcellular localizations of UMOD^C126R^ compared to UMOD^WT^ across the secretory pathway with greater precision, we utilized iterative indirect immunofluorescence imaging (4i), a technique that elutes antibodies cyclically so that many more proteins can be visualized in the same cell than with traditional techniques (31) (Supplemental Figure 4). Whereas UMOD^WT^ exhibited a predominantly membrane-localized pattern, UMOD^C126R^ accumulated intracellularly, as evidenced by its colocalization with Sec13 (COPII vesicles), COPB2 (COPI vesicles), and TMED2 and TMED9 (COP-Golgi) (Figure 1, D and E). These data showed that mutant UMOD was retained in the early secretory pathway, including TMED-enriched compartments.

### Targeting TMEDs reduces mutant UMOD in vivo and promotes its forward trafficking in vitro.

**Figure 2:**
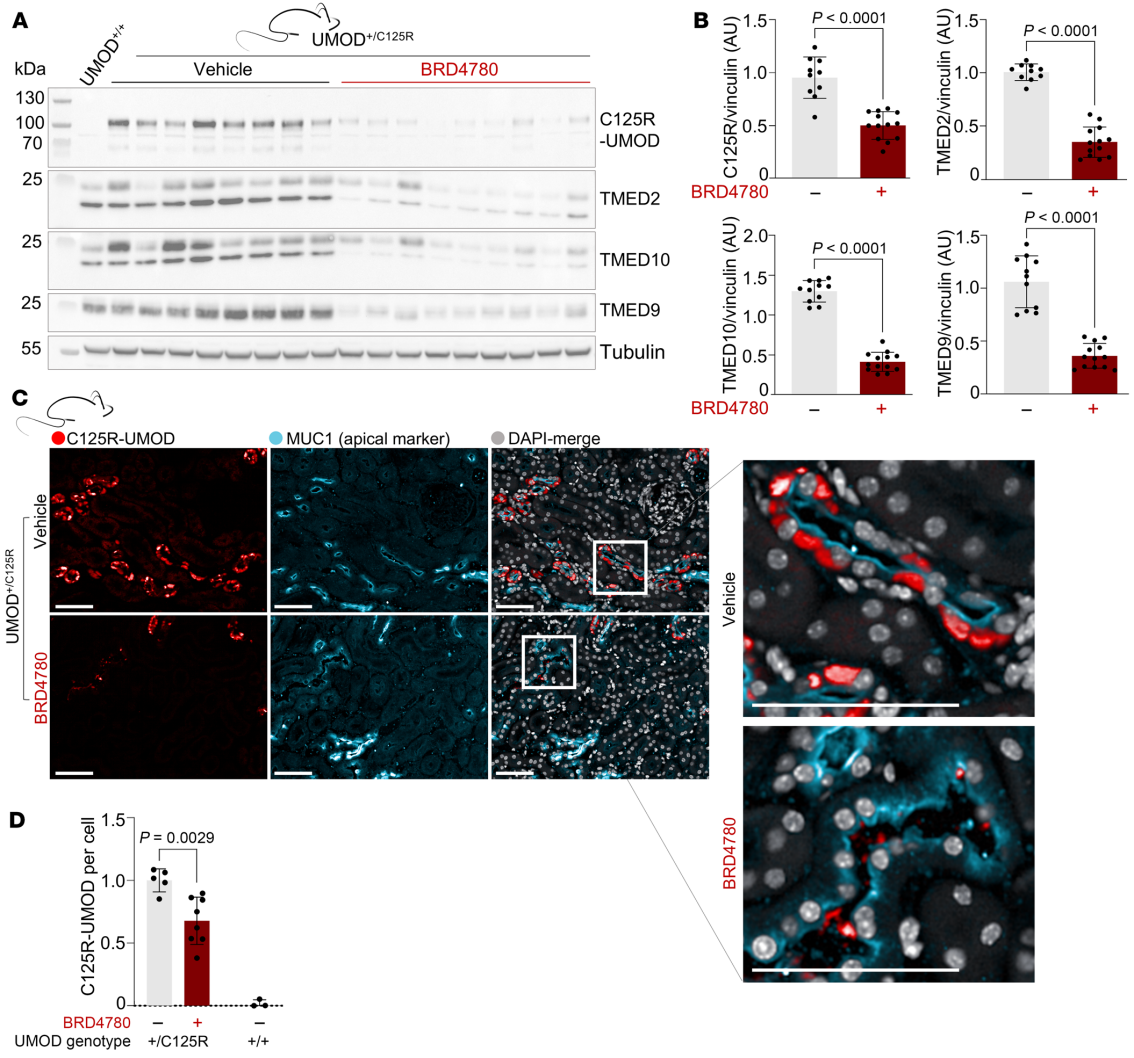
Targeting TMEDs reduces mutant UMOD accumulation in vivo. (**A**) Western blot of whole-kidney lysates from 10-month-old WT (UMOD^+/+^) and heterozygous knockin (UMOD^+/C125R^) mice treated with vehicle or BRD4780 (30 mg/kg) for 28 days. Blots were probed with a mutant-specific antibody (C125R-UMOD), or antibodies for TMED2, TMED10, and TMED9. (**B**) Densitometric analysis of the results in **A**. Statistical analysis was by unpaired 2-tailed *t* tests; data shown as mean ± SD, with each data point representing 1 mouse. (**C**) Immunofluorescence images of kidney sections from UMOD^+/C125R^ mice treated as above and stained for C125R-UMOD (red), the apical membrane marker MUC1 (cyan), and with DAPI (gray). All scale bars: 50 μm. Insets highlight tubular mutant UMOD clearance. (**D**) Quantitation of C125R signal in mouse cortex, outer and inner medulla, in MUC1^+^ cells, analyzed via 2-way ANOVA with Bonferroni’s post hoc test. Data shown as mean ± SD, with each data point representing 1 mouse.

Since TMEDs interact with UMOD, we sought to assess the effect of pharmacologically targeting TMEDs on the localization of UMOD. We therefore treated UMOD^+/C125R^ mice with BRD4780 (30 mg/kg) for 28 days. We also developed a custom-made C125R-specific antibody (Figure 2A, compare UMOD^+/+^ to UMOD^+/C125R^) to monitor levels of mutant UMOD^C125R^. Western blotting of whole-kidney lysates revealed a significant reduction in levels of TMED2, TMED9, and TMED10 after BRD4780 treatment, confirming target engagement by the drug (Figure 2, A and B). BRD4780 treatment also reduced the accumulation of mutant UMOD^C125R^ (Figure 2, A and B). These results in vivo support the hypothesis that TMED-UMOD^C125R^ interaction facilitates its entrapment. To further validate this observation, we performed immunofluorescence microscopy to visualize the effect of BRD4780 on the intracellular distribution of mutant UMOD. BRD4780 treatment resulted in partial clearance of accumulated mutant UMOD (Figure 2, C and D), in agreement with the Western blot results.

**Figure 3:**
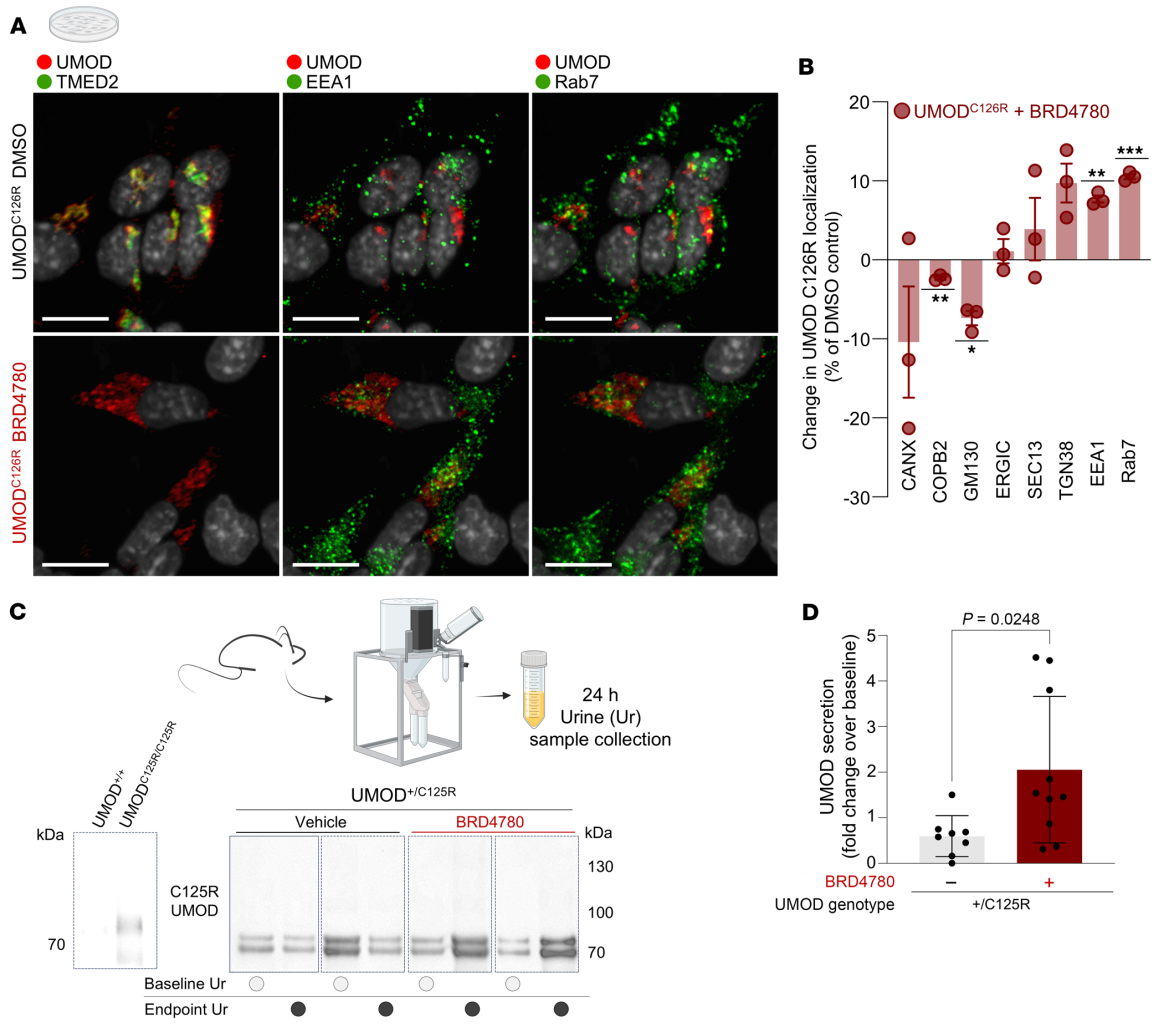
Targeting TMEDs promotes forward trafficking of mutant UMOD in vitro and urinary secretion in vivo. (**A**) Immunofluorescence images of AtT-20 cells stably transfected with mutant UMOD (UMOD^C126R^), treated with 5 μM BRD4780 or DMSO for 24 hours, and then processed using the 4i protocol. Scale bars: 10 μm. (**B**) Quantification of **A**. Colocalization is represented as change induced by BRD4780 compared with DMSO. Data shown as mean ± SEM from *n* = 3 technical replicates, analyzed by 1-sample *t* test against a theoretical mean of 0 (COPB2, *P* = 0.0069; GM130, *P* = 0.0147; EEA1, *P* = 0.0032; RAB7, *P* = 0.0009). Values per well represent averages from all the cells in a well (well 1: 461 cells; well 2: 911 cells; well 3: 973 cells). (**C**) Western blots of precipitated protein from urine of heterozygous knockin 10-month-old mice (UMOD^+/C125R^ mice) treated with vehicle or BRD4780 (30 mg/kg) for 28 days, detecting mutant-specific C125R-UMOD. The Western blot at the far left shows specificity of the C125R-specific antibody in whole-kidney lysates from WT (UMOD^+/+^) and homozygous knockin (UMOD^C125R/C125R^) mice. (**D**) Densitometric analysis of **C**. All urine samples were normalized to their baseline secretion to calculate secretion ratios. Statistical analysis by unpaired 2-tailed *t* test; data shown as mean ± SD, with each data point representing 1 mouse. *P < 0.05, **P < 0.01, ***P < 0.001.

Having established BRD4780’s efficacy in reducing the accumulation of mutant UMOD^C125R^, we sought to further clarify the mode of BRD4780’s action using 4i in vitro. BRD4780 treatment of UMOD^C126R^ cells reduced TMED2 levels (Figure 3A) and increased the colocalization of mutant UMOD^C126R^ with endosomal-lysosomal markers EEA1 and Rab7. We concluded that BRD4780 treatment promoted the anterograde trafficking of mutant UMOD into compartments of the late secretory pathway, including the lysosome (Figure 3, A and B). To verify that the loss of TMEDs promotes anterograde trafficking of mutant misfolded UMOD, we complemented our pharmacological approach with genetic disruption of TMED9 using CRISPR/Cas9. We performed 4i in these cells and found that genetic deletion of TMED9 phenocopied BRD4780’s promotion of mutant misfolded UMOD forward trafficking into the late secretory pathway, as quantified by decreased colocalization with COPB2 and increased colocalization with Rab7 (Supplemental Figure 5).

To facilitate the visualization of the colocalization dynamics of the mutant protein with lysosomes in real time, we performed live cell imaging following transient transfection of HEK293T cells with UMOD^C126R^ fused to mScarlet alongside a lysosomal GFP-tagged protein, TMEM192. This dual fluorescence live imaging approach revealed a BRD4780-dependent increase in colocalization of mutant protein and the lysosomal marker, in agreement with our 4i studies (Supplemental Figure 6).

We also hypothesized that, in vivo, increased anterograde traffic might mean that BRD4780 facilitates mutant UMOD secretion into the urinary space. To test this hypothesis, we precipitated proteins from urine of UMOD^+/C125R^ mice treated with either vehicle or BRD4780. We observed a significantly increased concentration of mutant UMOD in urine samples of BRD4780-treated mice, as assessed with the C125R-specific antibody (Figure 3, C and D). This experiment indicated that treatment with a TMED-targeted small molecule increased urinary secretion of mutant UMOD^C125R^.

### Targeting TMEDs restores UMOD^WT^ abundance on the apical plasma membrane.

**Figure 4:**
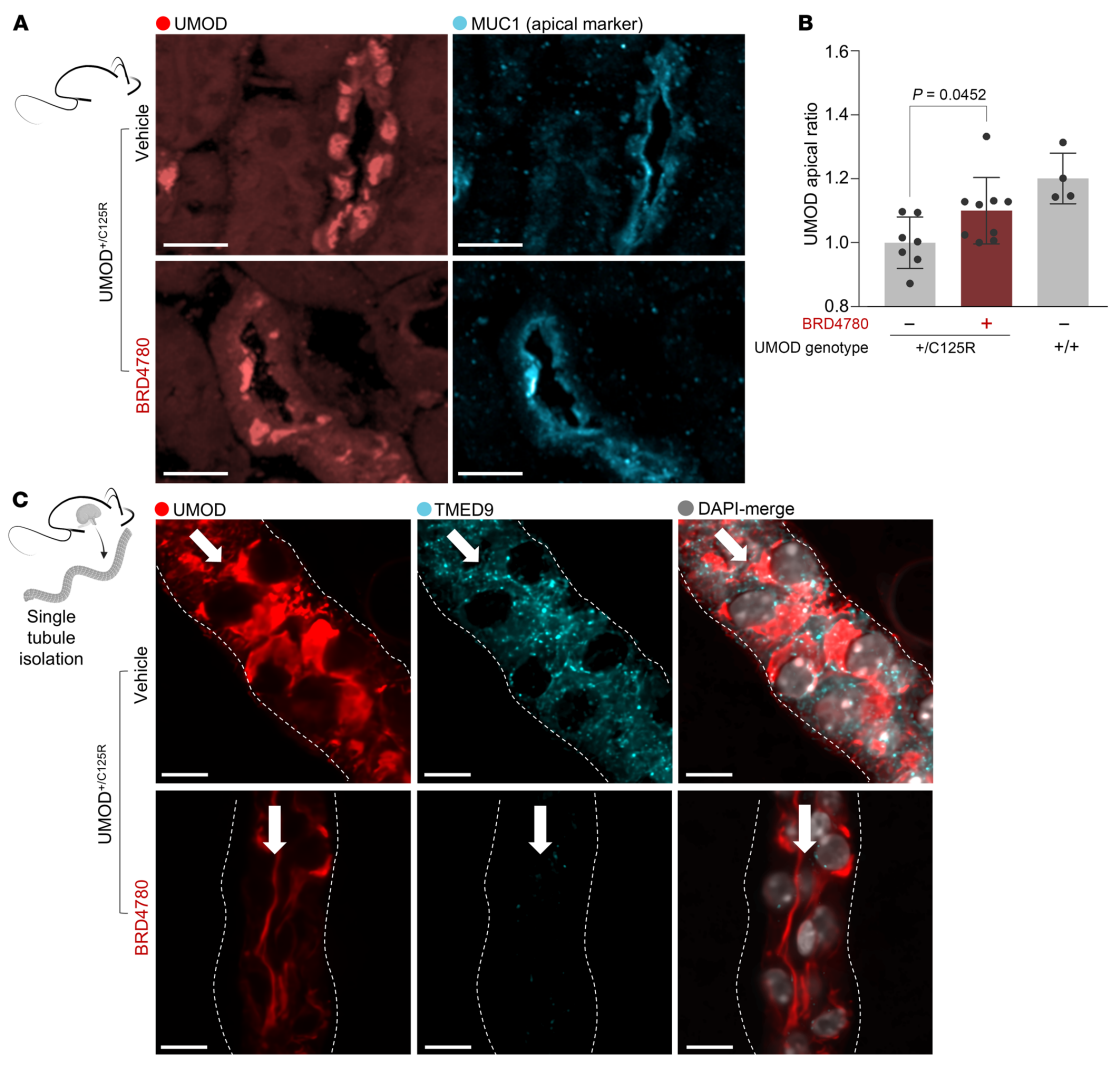
Targeting TMEDs restores WT UMOD localization to the apical epithelial plasma membrane. (**A**) Immunofluorescence images of kidney sections from heterozygous knockin (UMOD^+/C125R^) mice treated with vehicle or BRD4780 (30 mg/kg) for 28 days, stained for UMOD (red) and the apical membrane marker MUC1 (cyan). Scale bars: 25 μm. (**B**) Quantitation of UMOD distribution in MUC1^+^ cells within the cortex, expressed as the UMOD apical ratio (UMOD in MUC1 area/UMOD in intracellular area); analyzed via 2-way ANOVA with Bonferroni’s post hoc test. Data shown as mean ± SD, with each data point representing 1 mouse. (**C**) Immunofluorescence of single isolated TAL tubules from UMOD^+/C125R^ mice treated as above, stained for UMOD (red), TMED9 (cyan), and with DAPI (gray). Scale bars: 10 μm. White arrows indicate the tubular lumen, and the dotted lines represent the basolateral membrane.

In both mice and patients with UMOD mutations, apical membrane trafficking and secretion of UMOD is markedly impaired (32, 33). We reasoned that intracellular mutant UMOD accumulation leads to a “clogged” secretory pathway with impaired anterograde flow (as shown in Figures 2 and 3), and due to a dysfunctional secretory pathway, delivery of UMOD^WT^ to the apical plasma membrane might be reduced. To test this hypothesis, we asked whether BRD4780 treatment of heterozygous UMOD^+/C125R^ mice could restore the hallmark localization of UMOD to the apical membrane, crucial to maintenance of TAL cell polarity and function (4, 34, 35). We performed confocal immunofluorescence microscopy in mouse kidney tissue using an antibody that recognizes both UMOD^WT^ and mutant UMOD. We found that intracellular UMOD in kidney sections of heterozygous UMOD^+/C125R^ mice was markedly higher than in WT mice, especially in the perinuclear area, and that colocalization of UMOD with apical plasma membrane markers, such as MUC1, was decreased (Figure 4, A and B). BRD4780 treatment restored UMOD colocalization with MUC1 at the apical membrane (Figure 4, A and B) and reduced intracellular mutant UMOD accumulation (Figure 2C).

To analyze UMOD localization at higher resolution while maintaining tissue context, we isolated single tubule TAL segments and performed immunofluorescence microscopy (36–38). In this ex vivo system, we found a large portion of UMOD accumulated intracellularly in isolated tubules of UMOD^+/C125R^ mice. Targeting TMEDs with BRD4780 not only cleared the intracellular UMOD accumulation, but also restored levels of UMOD at the apical plasma membrane (Figure 4C). TMED9 was effectively reduced by BRD4780, confirming target engagement by the compound (Figure 2A and Figure 4C).

### Targeting TMEDs mitigates UMOD kidney disease.

Kidney accumulation of mutant, misfolded proteins such as UMOD^C126R^ and MUC1-fs results in activation of the UPR, which upregulates chaperones and reduces global protein translation, mechanisms aimed at reducing the misfolded protein load (39). In line with this model, GRP78 (also known as binding immunoglobulin protein [BiP]) is upregulated in kidney biopsies from ADTKD-*UMOD* patients (40). UPR proteins upregulated in TAL cells of mutant-UMOD mice include BiP, XBP1-s, and ATF4 (17, 25). Sustained ER stress leads to TAL cell apoptosis, initiating profibrotic pathways (16, 17, 19, 25). We therefore examined the potential effects of BRD4780 on ER stress, UPR, apoptosis, and kidney fibrosis.

**Figure 5:**
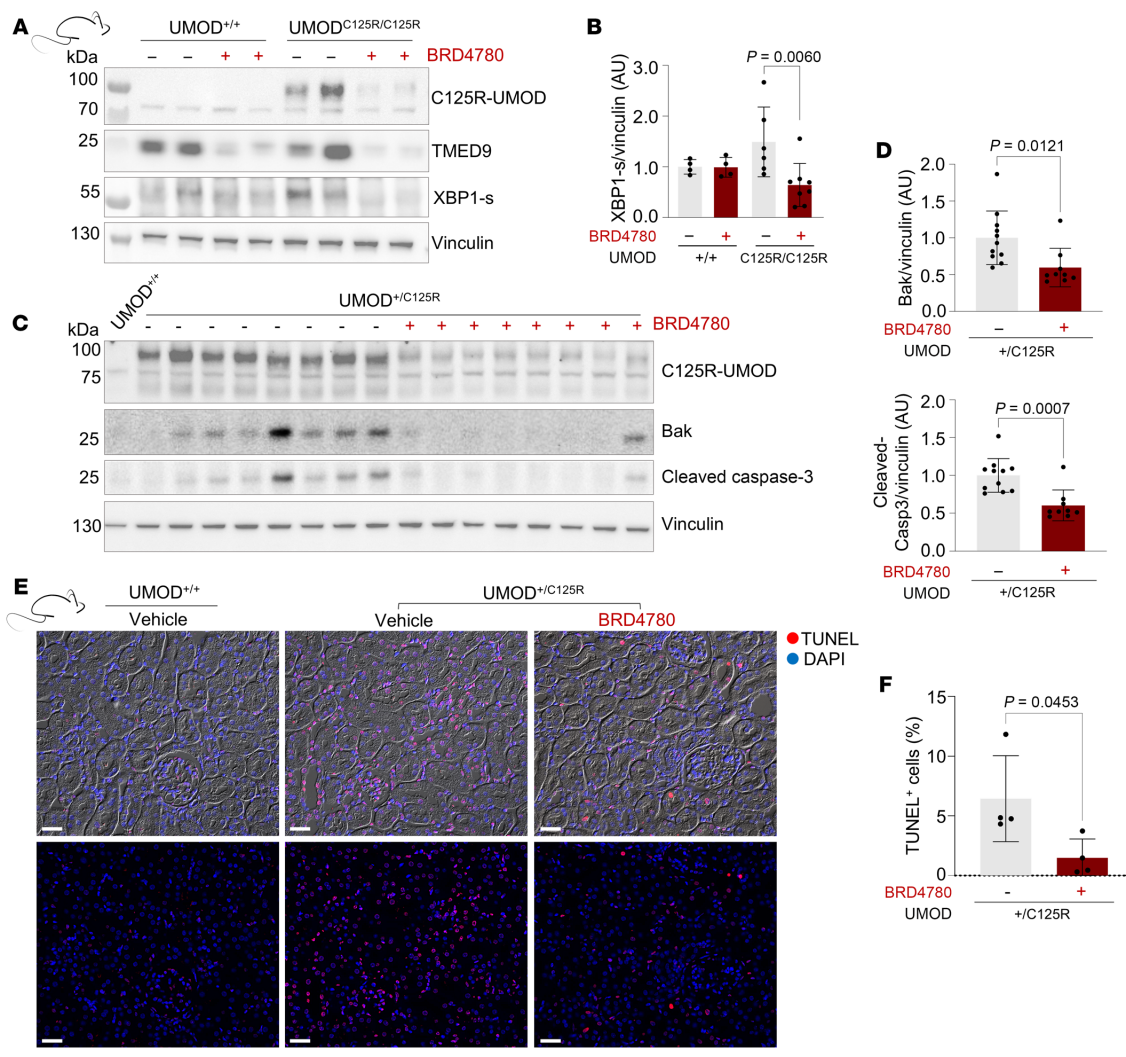
Therapeutic targeting of TMEDs protects tubular epithelial cells from ER stress and apoptosis. (**A**) Western blots of whole-kidney lysates from WT (UMOD^+/+^) and homozygous knockin (UMOD^C125R/C125R^) mice treated with vehicle or BRD4780 (30 mg/kg) for 28 days. (**B**) Densitometric analysis of **A**. Data were normalized to the mean values of the vehicle-treated UMOD^C125R/C125R^ mice and analyzed via 2-way ANOVA with Bonferroni’s post hoc test. Data shown as mean ± SD, with each data point representing 1 mouse. (**C**) Western blots of whole-kidney lysates from 1 UMOD^+/+^ mouse and heterozygous knockin (UMOD^+/C125R^) mice treated as above. (**D**) Densitometric analysis of **C**. Data were normalized to the mean values of the vehicle-treated UMOD^+/C125R^ mice and analyzed by Mann-Whitney test. Data shown as mean ± SD, with each data point representing 1 mouse. (**E**) Immunofluorescence images of TUNEL-stained kidney cortex from 10-month-old UMOD^+/+^ and UMOD^+/C125R^ mice treated as above. Top panel: Brightfield is shown as the background, TUNEL^+^ cells are shown in red, and DAPI is shown in blue. Bottom panel: Same image as above without brightfield. Scale bars: 20 μm. (**F**) Quantification of TUNEL^+^ cells from at least 3 cortical fields per mouse analyzed by unpaired 2-tailed *t* test; data shown as mean ± SD, with each data point representing 1 mouse.

We first asked whether targeting TMEDs and clearing intracellular UMOD accumulation would ameliorate ER stress. Western blot analyses of whole-kidney lysates from BRD4780-treated mice demonstrated a significant reduction in XBP1-s activation after treatment (Figure 5, A and B). Moreover, these lysates indicated lower expression levels of apoptotic markers, specifically cleaved caspase-3 and Bak, compared with vehicle controls (Figure 5, C and D). We also performed a TUNEL assay to corroborate the changes in apoptotic protein abundance in kidney tissue sections derived from WT mice, UMOD^+/C125R^ mice treated with vehicle, or UMOD^+/C125R^ mice treated with BRD4780. The UMOD^+/C125R^ mice that did not receive the therapeutic intervention exhibited a significantly higher number of TUNEL-positive cells, indicative of increased apoptotic activity. Treatment with BRD4780 resulted in a marked reduction in TUNEL-positive cells (Figure 5, E and F).

**Figure 6:**
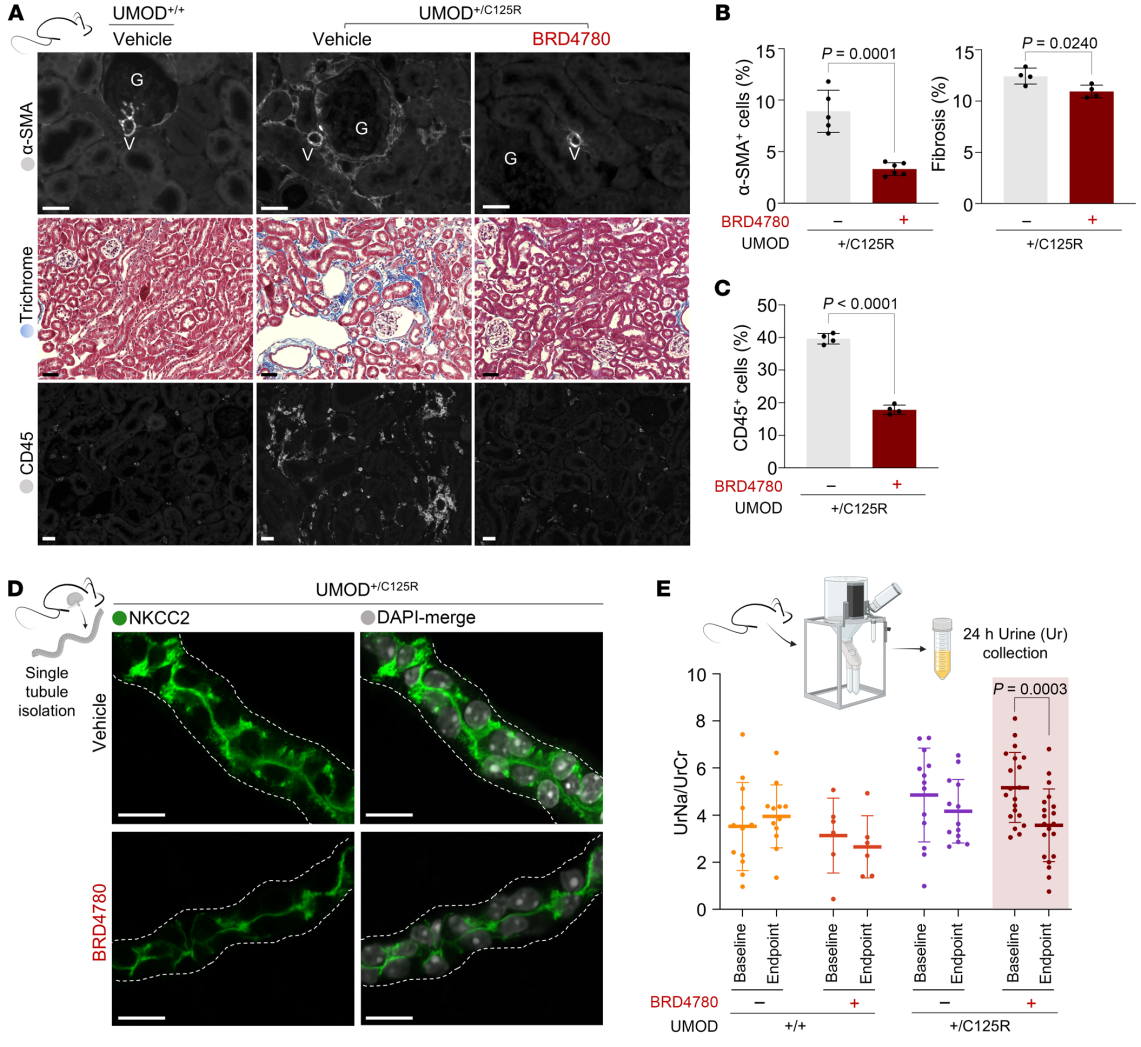
Therapeutic modulation of TMEDs mitigates inflammatory infiltrates and tissue fibrosis, restoring functional sodium reabsorption in the kidney. (**A**) Histological and immunofluorescence analysis of kidney sections from WT (UMOD^+/+^) and heterozygous knockin (UMOD^+/C125R^) mice treated with vehicle or BRD4780 (30 mg/kg) for 28 days. Sections were subjected to the following analyses: Top: α-Smooth muscle actin (α-SMA) immunofluorescent staining shown in gray. Scale bars: 20 μm. Middle: Masson’s trichrome staining showing collagen deposition (blue) indicative of fibrotic pathology within the renal interstitium. Scale bars: 40 μm. Bottom: CD45 immunofluorescent staining revealing leukocyte infiltration. CD45^+^ cells are shown in gray. Scale bars: 20 μm. (**B**) Quantitative analysis of fibrosis from at least 3 fields per mouse and analyzed by unpaired 2-tailed *t* test; data shown as mean ± SD, with each data point representing 1 mouse. Left: Quantitative assessment of α-SMA signal in the renal cortex. Right: Quantification of fibrotic areas via Masson’s trichrome staining, expressed as percentage (blue area/red area). (**C**) Quantification of CD45^+^ cells expressed as a percentage of total cells per field, from at least 3 fields per mouse and analyzed by unpaired 2-tailed *t* test; data shown as mean ± SD, with each data point representing 1 mouse. (**D**) Immunofluorescence imaging of isolated TAL tubules from UMOD^+/C125R^ mice treated as above: NKCC2 (green) and nuclei with DAPI (gray). Scale bars: 20 μm. The white dotted line indicates the basolateral membrane. (**E**) Assessment of urinary electrolyte handling and 24-hour urinary sodium excretion after treatment with BRD4780. Each data point represents an individual mouse, with paired samples between baseline and endpoint indicated; linking lines were omitted for clarity. Statistical analysis was conducted using 2-way ANOVA followed by Tukey’s multiple-comparison test.

Turning our attention to the downstream pathological implications of apoptosis, we next assessed the extent of tissue fibrosis. Immunofluorescent staining of kidneys for α-smooth muscle actin (α-SMA, an established marker of activated fibrogenic cells) and Masson’s trichrome staining revealed a significant decrease in fibrotic areas and collagen deposition after 28 days of BRD4780 treatment compared with vehicle-treated animals (Figure 6, A and B). Based on our previous work in mutant-UMOD mouse tissue that revealed abundant inflammatory infiltrates (41), we assessed the effect of BRD4780 on the abundance of CD45^+^ cells. The BRD4780-treated group demonstrated a significant reduction in inflammatory infiltrates, as evidenced by decreased CD45^+^ cell infiltration from nearly 40% down to 15% of all cells (Figure 6A, Figure 6C, and Supplemental Figure 7A). PAS staining also revealed improved overall kidney architecture after treatment with BRD4780 (Supplemental Figure 7B).

Finally, we investigated BRD4780’s effects on hallmark TAL parameters such as Na^+^/K^+^/2Cl^–^ (NKCC2) cotransporter localization. In dissected TAL tubule segments of UMOD^+/C125R^ mice, NKCC2 was detected in the apical plasma membrane, but also widely scattered intracellularly (Figure 6D). In sharp contrast, TAL segments from UMOD^+/C125R^ mice treated with BRD4780 retained abundant apical membrane localization while showing reduced intracellular accumulation of NKCC2 (Figure 6D), similar to BRD4780’s effects on UMOD (Figure 4C). Moreover, urinary sodium levels (partially dependent on functional NKCC2 in the TAL apical plasma membrane) in BRD4780-treated UMOD^+/C125R^ mice were decreased to levels comparable to WT mice (Figure 6E). This result is compatible with a beneficial restoration of epithelial cell health manifested as improved sodium reabsorption, likely through the reestablished apical membrane localization of NKCC2. Normalization of urinary sodium concentrations also served as an important in vivo functional measurement, confirming the beneficial salutary effects of small molecule treatment.

**Figure 7:**
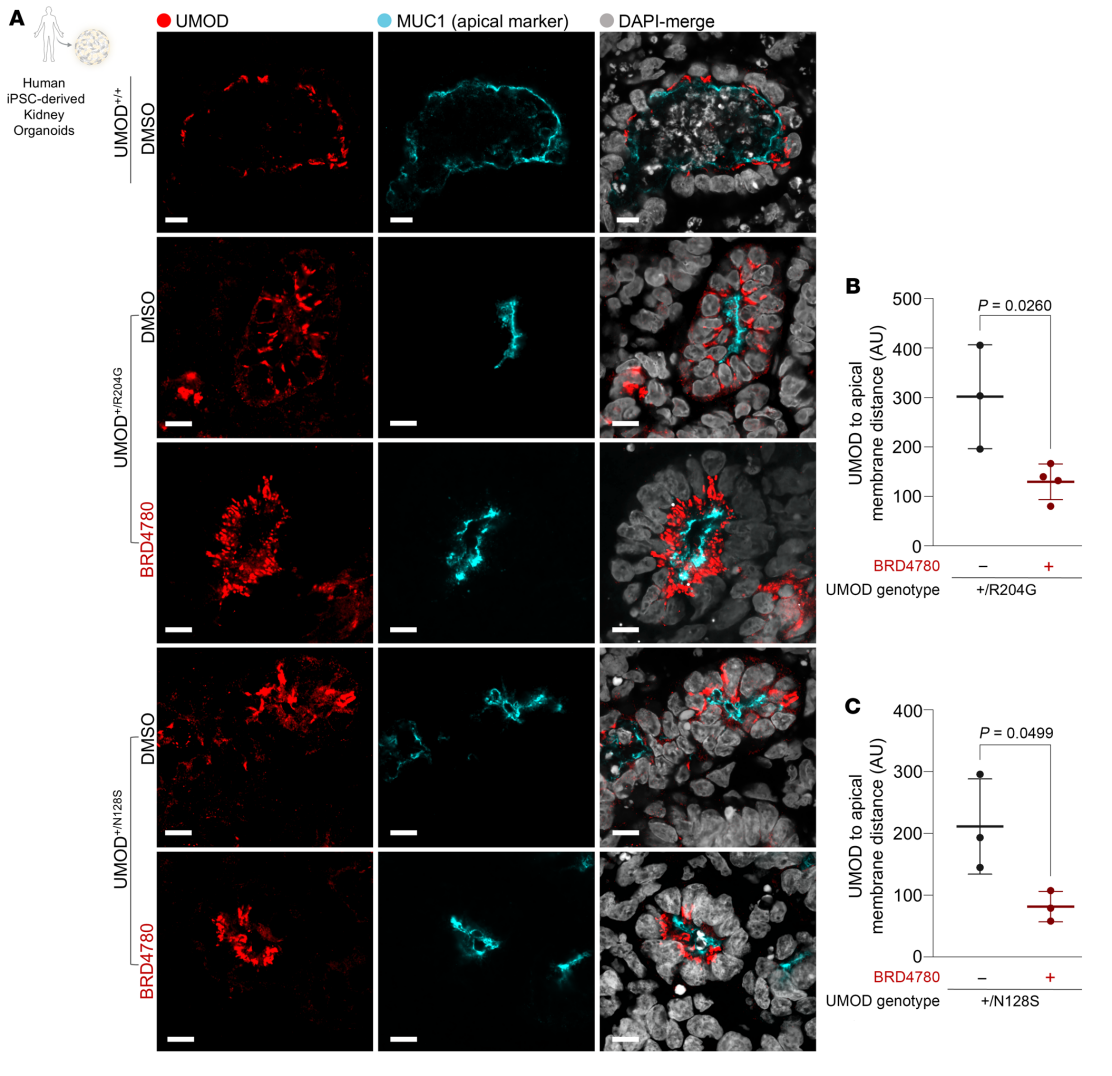
TMED-targeting compound removes intracellularly accumulated UMOD and restores apical localization of UMOD in human kidney organoids. (**A**) Immunofluorescence images of kidney organoids derived from 2 ADTKD-*UMOD* patients and 1 healthy donor (UMOD^+/+^). Patient 1 harbors the p.R204G mutation (UMOD^+/R204G^), and Patient 2 the p.N128S mutation (UMOD^+/N128S^). Organoids were treated with either DMSO (control) or 10 μM BRD4780 for 72 hours. Scale bar: 10 μm. (**B** and **C**) Quantitative analysis of the spatial localization between UMOD and the apical membrane marker MUC1, assessing the distance between these signals. Each data point represents the mean of analyses from 3–4 organoid tubules. Data were analyzed by unpaired 2-tailed *t* test and are presented as mean ± SD.

In a concerted effort to thoroughly investigate the therapeutic potential of BRD4780 in human cell models and across a broader spectrum of UMOD mutations, we developed kidney organoids cultured from human induced pluripotent stem cells (hiPSCs) harboring UMOD mutations distinct from C126R (20, 42). These organoids with Arg204Gly and Asn128Ser UMOD mutations exhibited abnormal UMOD intracellular accumulation and mislocalization of apical UMOD (Figure 7). Following treatment with BRD4780, detailed quantitative immunofluorescence analysis revealed a remarkable increase in the proximity between the apical membrane marker MUC1 and UMOD, suggesting effective restoration of UMOD to its physiological apical membrane localization, as opposed to the dispersed cytoplasmic distribution observed in untreated organoids (Figure 7). These results collectively underscore the efficacy of BRD4780 in rectifying protein mislocalization and restoring cellular homeostasis in human-derived kidney organoids.

## Discussion

In this study, we investigated mechanisms that regulate the trafficking of both WT and mutant UMOD. Our combined in vitro, ex vivo, and in vivo studies in mouse and human models showed that UMOD is bound by TMED cargo receptors 2, 9, and 10. We have further shown that treatment with a TMED-targeted molecule ameliorated the disease phenotype in 2 ways: it mitigated the toxic accumulation of the mutant protein, while also restoring UMOD to the apical plasma membrane. Our findings have several important implications.

Most ADTKD-*UMOD* mutations occur in exons 4 and 5 encoding EGF-like domains II and III, and the D8C domain of the protein — patients with UMOD mutations in these domains progress to kidney failure at a younger age (43). Many of our studies here were focused on a classical cysteine UMOD mutation, Cys126Arg, which resides in EGF-like domain III; however, we found that therapeutically targeting the TMEDs can also resolve entrapment for 2 other non-cysteine UMOD mutations, Arg204Gly and Asn128Ser, which are located in the cysteine-rich domain (D8C) and EGF-like domain III, respectively. How the location of the missense mutation affects the risk of developing severe kidney disease remains to be understood. We speculate that these domains represent important scaffold regions for binding to TMED GOLD domains and/or additional proteins involved in the complex process of UMOD trafficking. Based on this hypothesis, by directly targeting the entrapment of mutant UMOD by TMEDs, small molecules like BRD4780 may be widely applicable across the spectrum of pathogenic UMOD variants for the benefit of all patients with ADTKD-*UMOD*.

From a therapeutic standpoint, our study of multiple stress, apoptotic, fibrotic, and morphological markers supports the development of TMED-targeted small molecules as a potential treatment strategy for ADTKD-*UMOD*. Since (a) no treatments are currently available for this disease, and (b) ADTKD-*UMOD* and ADTKD-*MUC1* appear to share TMEDs as a common, nodal biological pathway underlying both diseases, our study provides the rationale for deployment of this therapeutic strategy to both patient populations. Current estimates suggest that there are more than 100,000 patients with ADTKD in the United States alone (44). In a United Kingdom cohort, ADTKD-*UMOD* accounted for 9% of all inherited kidney diseases, and strikingly, it accounted for 1% of all stage 3–5 chronic kidney disease (CKD) (45, 46). Thus, testing this therapeutic hypothesis in the clinic could benefit a significant subset of patients whose kidneys fail due to the lack of any currently available therapies.

Our studies showed that UMOD interacts with TMED cargo receptors TMED2, TMED9, and TMED10, implicating this family of proteins in the quality control processes that lead to toxic ER retention of misfolded UMOD, a GPI-anchored protein. BRD4780, a small molecule compound targeting TMEDs, was an effective tool for these mechanistic studies in several in vitro, ex vivo, and in vivo models. Additionally, our work on organoids and isolated single TAL tubules enabled mechanistic studies of endogenously expressed UMOD under controlled and reproducible conditions. Organoids have the advantage of being easily derivable from human cells, whereas isolated tubules preserve the native structure of the nephron. We expect that organoid differentiation and the isolated-tubule approach will continue to be powerful tools for future UMOD biology research.

Finally, beyond monogenic ADTKD-*UMOD*, seminal genome-wide association studies (GWAS) conducted in different CKD populations around the world have identified UMOD as the most significant genetic locus associated with CKD (47–49). Common genetic variants residing in the *UMOD* promoter region are associated with altered UMOD expression (50) and, through yet unknown pathogenic mechanisms, elevated blood pressure (51) and decreased kidney function (52). A recent pivotal study has also found intermediate-effect size variants of UMOD-related pathology, manifesting in attenuated forms of renal disease (53). These findings suggest a spectrum of UMOD-associated disorders, broadening the traditional dichotomy between severe autosomal dominant diseases and subclinical manifestations. The discovery of these intermediate variants underscores the complexity of UMOD’s role in kidney pathology and highlights the need for a nuanced understanding of genotype-phenotype correlations. As CKD is a global health crisis affecting approximately 10% of the human population (54, 55), new, mechanism-based therapies as described here are urgently required to address the enormous unmet need. In sum, our work reveals TMED-targeting small molecules as a promising therapeutic strategy for kidney proteinopathies, and may inspire future studies aimed at exploring CKD through the lens of cellular responses to altered protein homeostasis.

## Methods

### Sex as a biological variable

For studies involving humans and/or animal models, sex was not considered as a biological variable. Our study exclusively examined male mice. It is unknown whether the findings are relevant for female mice.

### Cell culture

HEK293T cells (ATCC) were cultured at 37°C in 5% CO_2_ in Dulbecco’s modified Eagle’s medium (DMEM), high glucose, GlutaMAX (Gibco) supplemented with 10% fetal bovine serum (FBS) (Invitrogen) and 1% penicillin/streptomycin (Life Technologies). AtT-20 cells (ATCC) stably transfected with cDNAs encoding UMOD^WT^ or UMOD^C126R^ were cultured (20) at 37°C with 5% CO_2_ in DMEM/F12 (1:1) supplemented with 10% FBS, 0.5% penicillin/streptomycin, and 0.8 mg/mL Geneticin 418 (Roche). All cell lines were routinely checked for mycoplasma.

### Mutagenesis and constructs

FLAG-tagged UMOD^WT^ in pcDNA3.1+/C-(k)DYK (clone ID OHu21261) was purchased from GenScript. Mutant UMOD construct c.385T>C (C126R-UMOD) was prepared by site-directed mutagenesis (QuikChange, Stratagene) of the UMOD^WT^ construct, using custom-made primers (IDT). Engineered mutations were confirmed by DNA sequencing.

### Generation of TMED9-knockout cell line

To generate TMED9-knockout (TMED9-KO) cells, sgRNA targeting *TMED9* was designed using Dharmacon’s online CRISPR RNA design tool (Dharmacon/GE Healthcare). The chosen sgRNA sequence (GCCCGGCAGTATGGCTCTGA for Entrez Gene 67511) was cloned into the All-in-one lentiviral vector, which contains both sgRNA and Cas9 nuclease under the control of the U6 and EF1α promoters, respectively.

### Lentiviral infection

AtT-20 cells stably transfected with C126R-UMOD were transduced with the Dharmacon All-in-one lentiviral vector. Polybrene (MilliporeSigma) was added to the viral suspension at a final concentration of 8 μg/mL to enhance transduction efficiency. Target cells were then exposed to the viral suspension and incubated at 37°C with 5% CO_2_. Following 24-hour incubation, the viral supernatant was replaced with fresh culture medium.

### Selection and expansion

Puromycin (Thermo Fisher Scientific) was added to the culture medium at a concentration of 1.5 μg/mL, and selection was maintained for the rest of the experiments. TMED9 KO was validated with immunofluorescence (Supplemental Figure 5C).

### Cell transfection and lysis for co-IP

HEK293T cells were transiently transfected with UMOD^WT^ or UMOD^C126R^ using Lipofectamine 2000 (Life Technologies) following the manufacturer’s instructions. Culture dishes (10 cm) were seeded at 80% confluence. The next day, cells were transfected with 8 μg/plate of total plasmid DNA (UMOD^WT^ or UMOD^C126R^) in a 2:1 ratio with Lipofectamine 2000 in Opti-MEM media (Gibco) and incubated overnight. The next day, fresh media were added. Forty-eight hours after transfection, cells were washed with ice-cold 1× PBS and then lysed with 800 μL/plate of NP-40 lysis buffer (100 mM NaCl, 5 mM EDTA, 50 mM Tris-HCl, 1% NP-40) containing protease inhibitors (Roche, 04693159001) and phosphatase inhibitors (Roche, 04906837001). Lysates were rotated 30 minutes at 4°C, and then centrifuged at 13,000*g* for 15 minutes at 4°C. Supernatants were transferred to new 1.5 mL Eppendorf tubes for protein measurement (Pierce BCA Protein Assay, Thermo Fisher Scientific). Protein (1 mg) was rotated overnight at 4°C with 20 μL of anti-FLAG M2 Magnetic Beads (Sigma-Aldrich) following the manufacturer’s instructions. Beads were washed once with NP-40 lysis buffer and twice more with TBS (150 mM NaCl, 50 mM Tris, pH 7.5). Washed beads were resuspended in a 30 μL elution buffer (2× NuPAGE LDS Sample Buffer, 1× NuPage Sample Reducing Agent; Thermo Fisher Scientific) and heated at 75°C for 10 minutes. Supernatants were separated on a magnetic rack, transferred to new tubes, and loaded on SDS-PAGE gels for Western blot (see “Cell protein extraction and Western blot analysis” below) or resuspended in 1 mL 1× TBS for IP-MS (see “IP-MS sample processing” below). TMED9-MYC co-IPs were performed using 1 mg of protein and 25 μL of Anti-c-Myc Magnetic Beads (Pierce). Samples were rotated 30 minutes at room temperature, washed 3 times with 1 mL 5× TBS containing 0.05% Tween 20, and once with 1 mL Milli-Q water before resuspension in elution buffer (2× NuPAGE LDS Sample Buffer, 1× NuPage Sample Reducing Agent). Samples were then heated at 75°C for 10 minutes, supernatants were separated on a magnetic rack, transferred to new tubes, and loaded on SDS-PAGE gels for Western blotting.

### Cell transfection for live imaging

We transfected (see “Cell transfection and lysis for co-IP”) plasmids UMOD[001378235]-C126R-Scarlet (VectorBuilder) and pLJM1-FLAG-GFP-Tmem192 (Addgene plasmid 134630; RRID: Addgene_134630) (56). Following the transfection-incubation period at 37°C with 5% CO_2_, the transfection medium was replaced with fresh complete growth medium. The next day, cells were seeded at 60%–70% confluence in glass-bottom CellCarrier Ultra microplates (384-well, PerkinElmer) precoated with 0.25 mg/mL Syn-themax II SC Substrate (Corning). Cells were incubated for an additional 24–48 hours to allow for optimal expression of the fluorescently tagged protein. Immediately prior to live imaging, the growth medium was replaced with phenol red–free DMEM (Gibco) supplemented with 10% FBS, 1% GlutaMAX, and 25 mM HEPES buffer (Gibco) to maintain pH stability and minimize autofluorescence. DMSO or 5 μM BRD4780 was added and cells were maintained at 37°C in a live-cell imaging chamber during the imaging sessions.

### Imaging procedure

Live-cell imaging was performed using the Opera Phenix High-Content Screening System (PerkinElmer). A minimum of 9 fields were acquired per well using 40× water immersion objectives in confocal mode. Time-lapse and high-resolution images were captured using appropriate excitation and emission settings for the fluorescent reporter. Image acquisition parameters were kept consistent across different experimental conditions to facilitate quantitative analysis.

### Cell BRD4780 treatment and lysis

Culture dishes (10 cm) were plated and grown 48 hours to confluence. Confluent cells were treated with 5 μM BRD4780 or an equivalent volume of DMSO vehicle (Sigma-Aldrich) for the indicated times. Treated cells were washed with ice-cold 1× PBS and lysed with 800 μL NP-40 lysis buffer. Lysates were rotated 30 minutes at 4°C, and then centrifuged at 4°C for 15 minutes at 13,000*g*. Supernatants were transferred to new 1.5 mL Eppendorf tubes for protein measurement (Pierce BCA Protein Assay). Clarified lysates were prepared for SDS-PAGE at 1 μg/μL in 1× NuPAGE LDS Sample Buffer and 1× NuPage Sample Reducing Agent and heated at 95°C for 10 minutes.

### Cell protein extraction and Western blot analysis

Protein (20 μg) was loaded in NuPAGE 4%–12% Bis-Tris gels with PagerulerPlus Preset M_r_ standards (Thermo Fisher Scientific) and electrophoresed 2 hours at 125 V in 1× MES-SDS buffer. Separated proteins were transferred to nitrocellulose membranes (Bio-Rad) using the Trans-Blot Turbo Blotting System (Bio-Rad) per manufacturer’s instructions. Membranes blocked in 10% nonfat dry milk (Cell Signaling Technology) in 1× PBS with 0.3% Tween 20 (PBS-T) were probed with primary antibodies (diluted in 5% milk in PBS-T) overnight at 4°C (see Supplemental Table 1 for list of antibodies). Probed membranes were washed 3 times in PBS-T for 10 minutes while shaking at room temperature, and then probed with secondary antibody (diluted in 5% milk in PBS-T) 1 hour at room temperature. Membranes were again washed 3 times for 10 minutes each with PBS-T before 5-minute incubation in SuperSignal West Pico Plus Chemiluminescent Substrate or (as specified) 3-minute incubation in SuperSignal West Femto Plus Chemiluminescent Substrate (Life Technologies) and imaged in chemiluminescent and colorimetric channels (ChemiDoc Imager, Bio-Rad, 12003154) with densitometric analysis (ImageJ software, NIH).

### Preparation of cells for 4i

We performed 4i as previously described (31), with minor modifications. A PerkinElmer 384-well PhenoPlate was coated with Corning Synthemax II-SC Substrate and then seeded with UMOD^WT^ AtT-20 and UMOD^C126R^ AtT-20 cells at a density of 7,000 cells per well. The plate was incubated at 37°C overnight to allow cells to attach. On day 1, treatment (DMSO + media or 5 μM BRD4780 + media) was administered, and the cells were left to incubate at 37°C for 24 hours. On day 2, the cells were fixed using a 4% paraformaldehyde (PFA) solution for 30 minutes at room temperature, with continuous shaking. Fixed cells were then washed 4 times with 1× PBS and permeabilized for 15 minutes at room temperature using a 1% Triton X-100/PBS solution (Triton X-100 Surfact-Amps, Thermo Fisher Scientific). Following 4 cycles of PBS and ddH_2_O washing, the cells were treated 3 times with elution buffer for 10 minutes at room temperature (0.5 M L-glycine, 3 M urea, 3 M guanidinium chloride, and 70 mM TCEP-HCl [all from Sigma-Aldrich] in ddH_2_O; pH 2.5). Cells were then washed with 4 cycles of PBS and immediately blocked using LI-COR Intercept blocking buffer (LI-COR Biosciences), supplemented with 150 mM maleimide for 1 hour at room temperature. Primary antibodies were diluted in blocking reagent (see Supplemental Table 1 for list of antibodies). The cells were incubated with antibodies for 1 hour at room temperature with continuous shaking, followed by an additional hour at 37°C. Secondary antibodies (see Supplemental Table 1) were prepared in blocking solution and applied at a dilution of 1:500, following washing with PBS. The cells were incubated for 1 hour at room temperature, immediately washed with PBS, then treated with 1:1,000 diluted 4′,6-diamidino-2-phenylindole (DAPI; Thermo Fisher Scientific) in PBS. The DAPI dilution was adjusted by a factor of 1.25× after each cycle due to potential degradation of nuclear contents. The cells were incubated for 10 minutes at room temperature, followed by 4 cycles of washing with ddH_2_O. Finally, an imaging buffer (700 mM *N*-acetyl-cysteine in ddH_2_O, pH 7.4), was added to each well. For each imaging cycle, the protocol was repeated, starting with application of the elution buffer solution.

### Cellular 4i analysis and computation

All 4i fluorescence imaging was performed using the Opera Phenix High-Content Screening System (PerkinElmer). A minimum of 25 fields with 5 *Z*-stacks were acquired per well using 63× water immersion objectives in confocal mode. Microscopy images were exported and processed using CellProfiler 4 (https://cellprofiler.org) and custom computational pipelines using Standard CP modules. First, images were loaded with appropriate metadata. Next, a Maximum Intensity Projection was applied to each set of images using the MakeProjection module. As noted in Gut et al. (31), images of different cycles from the same site shift slightly in *X* and *Y* between imaging sessions due to imperfect stage repositioning and require computational image alignment. Image registration was performed using the DAPI channels from all cycles of 4i. Cycles were aligned using the Normalized Cross Correlation algorithm and the images were cropped to the area of alignment using the Align CP module. The corresponding 488 nm and 568 nm channels were shifted by the calculated offset. Once the images were aligned, flat-field illumination correction was applied and features were extracted. The IdentifyPrimaryObjects module was used to segment nuclei from images of the DAPI channel following a 2-class Otsu thresholding method. Cellular segmentations using the IdentifySecondaryObject module were achieved by using the calnexin signal (Cycle 2) and employing a Minimum Cross-Entropy thresholding strategy. Finally, the Cytoplasm segmentation was computed using the IdentifyTertiaryObject module, which subtracted the Nuclear from the Cell segmentation. The FilterObjects module was used to remove cells that were in contact with the border of the image. Next, for each secretory marker measured, an object and binary mask were created using IdentifyPrimaryObject and then transformed into a mask using the ConvertObjectToImage and MaskImage modules. These new objects were related to their parent cell using the RelateObject module. Features were then extracted using standard CellProfiler morphology, colocalization, texture, and intensity measurement modules. Single-cell stain correlation measures (Pearson’s coefficients) between secretory markers were calculated and plotted in histograms to visualize the colocalization of secretory markers across all cycles. The measurement of UMOD levels within subcellular compartments was conducted by using binary representations of each stain and calculating the proportion of total UMOD overlapping with each compartment within each cell. Aggregated values across subcellular secretory compartments were compared between vehicle-treated and drug-treated samples.

### Mouse model

UMOD-knockin (UMOD-KI) mice were transferred to the Broad Institute from the Thakker lab at the University of Oxford (25). UMOD-KI heterozygous mice were mated to produce the WT (UMOD^+/+^), UMOD-KI heterozygous (UMOD^+/C125R^), and homozygous (UMOD^C125R/C125R^) progeny used in these studies. Mice were maintained on a 12-hour light/dark cycle in an AAALAC-accredited facility with ad libitum access to water and standard rodent chow.

### BRD4780 treatment of UMOD-KI mice

The effect of BRD4780 on mouse kidneys was tested in age-matched (9–10 months) male UMOD^+/+^, UMOD^+/C125R^, and UMOD^C125R/C125R^ mice. BRD4780 (30 mg/kg/day) or saline vehicle were administered daily by oral gavage for 28 days. Animal weight and behavior were observed daily. Mice were placed in metabolic cages before (baseline) and after treatment (endpoint) to collect 24-hour urine and blood samples for biochemical evaluation. At the end of the treatment period, mice were anesthetized with 3 L/min 3% isoflurane in O_2_ (Combi-vet system, Rothacher Medical). Anesthetized mice were intracardially perfused with PBS (pH 7.4) prior to resection of kidneys for histologic or immunoblot studies (see “Kidney histology and immunofluorescence” and “Kidney protein extraction and Western blot analysis”).

### Urine analysis

Urine samples were collected in 50 mL conical tubes, clarified by centrifugation at 4°C (2,000*g*, 15 minutes) and stored at –80°C. Urine samples were analyzed by The George M. O’Brien Kidney Center at Yale University School of Medicine.

### Kidney histology and immunofluorescence

PBS-perfused mice were perfused with 4% PFA. Resected kidneys were decapsulated and incubated at least 4 hours at room temperature in 4% PFA, and then overnight with rotation in 1% PFA, followed by dehydration in 30% sucrose. Kidneys for cryosection were transversally transected, mounted in OCT, and flash frozen in dry ice/ethanol. Decapsulated kidneys for histological study were immediately transferred to formalin-filled containers until processed for standard paraffin-embedding and sectioning. Sections (4 μm) were mounted on glass slides and deparaffinized. Deparaffinized sections were PAS stained, or epitope retrieval performed by 6-minute heating at 95°C in 10 mM Na citrate, pH 6.0. After 30-minute room temperature block in 1× TBS containing 5% BSA (Sigma-Aldrich), sections were incubated overnight at 4°C with primary antibody diluted in blocking solution (see Supplemental Table 1 for list of antibodies). After washing in 1× TBS, sections were incubated 1 hour at room temperature with fluorescently labeled secondary antibodies diluted in blocking solution and washed 3 times. Nuclei were stained with DAPI (Invitrogen) and slides were mounted in ProLong Gold Antifade Mountant (Thermo Fisher Scientific). For TUNEL assay, paraffin sections were deparaffinized as described above, and then incubated for 10 minutes at room temperature with 20 μg/mL Proteinase K in 10 mM Tris-HCl pH 8.0 (Roche, 03115828001). Subsequent steps were performed according to the manufacturer’s instructions (Roche, 11684795910). Confocal images were acquired on a Nikon Ti2E microscope with a motorized encoded stage using the 60×/1.40 oil objective. Additional images were collected with an AX Laser Scan Confocal with Resonance scan capabilities and laser lines 405, 488, 561, and 640 nm operated on Nikon AR software 5.42.02. Images were exported with ImageJ.

### Apicality analysis

Kidney cryosections (5 μm thick) were placed in a 24-well Sensoplate (Greiner Bio-One) coated with (3-aminopropyl)triethoxysilane (APTES). Sections were air dried for 10 minutes, washed 3 minutes in 1× PBS, blocked in 5% BSA (Sigma-Aldrich) in 1× PBS with 0.05% Triton X-100, and then incubated overnight at 4°C with primary antibodies diluted in blocking buffer (Armenian hamster anti-MUC1 and sheep anti-UMOD antibodies listed in Supplemental Table 1). Incubated sections were washed with 1× PBS, incubated in secondary antibodies (1:500 in blocking solution) for 2 hours at room temperature, incubated 15 minutes with DAPI (1:5,000 in 1× PBS), washed 3 times, and left in 1× PBS for imaging. For apicality analysis, tissue sections were imaged with the Opera Phenix High-Content Screening System (PerkinElmer) in 24-well glass bottom plates (1 kidney per well), using the “Preciscan” option in Harmony Software (PerkinElmer) to find sections on the plate using 5× scan and ×20 confocal mode for entire tissue image acquisition. Blurred DAPI signal was used for tissue segmentation, and the very peripheral edges were excluded. The cortex region was identified using the “find texture region” block in Harmony software, based on *Lotus* *tetragonolobus* lectin (LTL) (Alexa Fluor 488) signal. Papilla was sometimes included in this region, and we excluded it based on high intensity in the MUC1 channel. “Apical Area” of the MUC1 positive tubules was segmented using the “find image region” feature with a high threshold for MUC1 staining. To exclude intratubular space from analysis, “Image Region” with the low threshold MUC1 intensity was then segmented. “Tubular Area” was selected by expanding the apical area by 8 μm within MUC1 low signal “Image Region.” “Intracellular Area” was selected by excluding “Apical Area” from “Tubular Area.” Mean UMOD signal was calculated within the tubular area and cutoff was determined to select all UMOD-positive tubules. In the UMOD-positive tubules, mean UMOD signal was measured separately in the “Apical Area” (A) and in the “Intracellular Area” (B). Ratio A/B was calculated for each tubule and taken as an apicality measure, which was averaged for all tubules in a section.

### Kidney protein extraction and Western blot analysis

Kidneys were removed, decapsulated, and flash frozen in liquid nitrogen. One half of a transverse-cut kidney was lysed using a tissue homogenizer (Kinematica, PT1200 E) in 500 μL of NP-40 lysis buffer (100 mM NaCl, 5 mM EDTA, 50 mM Tris-HCl, 1% NP-40) containing protease inhibitors (Roche, 04693159001) and phosphatase inhibitors (Roche, 04906837001). The lysate was centrifuged at 14,000*g* and 4°C for 10 minutes and the supernatant was recovered. Lysate protein was measured (Pierce BCA Protein Assay Kit) and prepared (3 μg/μL) in 1× NuPAGE LDS Sample Buffer and 1× NuPage Sample Reducing Agent sample buffer prior to heating for 10 minutes at 95°C and performing SDS-PAGE using NuPAGE MES-SDS1X running buffer (Thermo Fisher Scientific) for 4%–12% gels or NuPAGE Tris-Acetate-SDS1X running Buffer for 3%–8% gels. Electrophoretically separated proteins were transferred to a nitrocellulose membrane (Bio-Rad) using the Trans-Blot Turbo Blotting System (Bio-Rad) per manufacturer’s instructions. Membranes were blocked in 10% nonfat dry milk in PBS-T and probed with primary antibody diluted in 5% milk in PBS-T overnight at 4°C. Following a 30-minute PBS-T wash, membranes were incubated 1 hour at room temperature with secondary antibodies, and then washed 1 hour in PBS-T, 5 minutes with 1× PBS, and incubated with Super Signal West Femto (Thermo Fisher Scientific) or Super Signal West Pico (Thermo Fisher Scientific) depending on the antibody. Chemiluminescent bands were imaged with a ChemiDoc Imager (Bio-Rad, 12003154). Densitometric analysis was performed with ImageJ. Full details of antibodies used are included in Supplemental Table 1. Membranes were then incubated with secondary anti-rabbit IgG HRP-linked (Cell Signaling Technology).

### Kidney endogenous co-IP

One kidney was homogenized in 1 mL of NP-40 lysis buffer using a tissue homogenizer (see “Kidney protein extraction and Western blot analysis”). Lysate containing 2 mg of protein was combined with 25 μL of THP (B-2) agarose beads (sc-271022, lot A3023, Santa Cruz Biotechnology) or normal mouse IgG conjugated agarose beads (sc-2343, lot I2722, Santa Cruz Biotechnology) as control. Samples were left rotating overnight at 4°C. The next day, beads were washed once with NP-40 lysis buffer and twice with 5× TBS with 0.05% Tween 20. Washes were performed by pelleting beads by centrifugation at 1,000*g* for 30 seconds at 4°C. Beads were then resuspended in 30 μL of elution buffer (2× NuPAGE LDS Sample Buffer, 1× NuPage Sample Reducing Agent), heated at 95°C for 10 minutes, and the supernatant was transferred to new tubes to load gels for further analysis by Western blot.

### TCA-acetone urine protein precipitation

One part TCA solution (10 g of TCA in 10 mL of Milli-Q water) was thoroughly vortexed with 4 parts of previously centrifuged urine following previous recommendations (9). The mixture was then incubated on ice for 1 hour and centrifuged at 11,000*g* for 10 minutes at 4°C. The supernatant was discarded, and the pellet was washed twice with 200 μL of cold acetone (–20°C). The washed pellet was dissolved in NP-40 lysis buffer and left overnight at –20°C to increase protein yield prior to protein measurement (Pierce BCA Protein Assay Kit), then prepared at 1 or 3 μg/μL in 1× NuPAGE LDS Sample Buffer/1× NuPage Sample Reducing Agent sample buffer for Western blot (see “Kidney protein extraction and Western blot analysis”).

### Enzymatic tubule dissection

Kidneys immediately removed from euthanized mice were minced to obtain an enzymatic tubule suspension (57). After decapsulation, very thin transverse slices were then transferred to a 2 mL Eppendorf tube containing incubation solution (140 mM NaCl, 0.4 mM KH_2_PO_4_, 1.6 mM K_2_HPO_4_, 1 mM MgSO_4_, 10 mM Na-acetate, 1 mM α-ketoglutarate, 1.3 mM Ca-gluconate, 25 mg/L DNase I, 375 mg/L glycine, and 48 mg/L trypsin inhibitor) and collagenase II (2 mg/mL) in a ThermoMixer (Eppendorf, 850 rpm, 37°C) to drive tubule dissociation. The dissociated tubules were placed in a small culture dish containing 4°C sorting solution (incubation solution plus 500 mg/L BSA) for tubule sorting. TALs were identified under a dissecting microscope and manually sorted. Approximately 10 TALs were placed directly on ice-cold Polylysine Adhesion Slides (Thermo Fisher Scientific) and prepared for immunofluorescence (57).

### Immunofluorescence of isolated tubules

TALs were fixed on microscope slides with 4% PFA for 7 minutes, and then thoroughly washed with 1× PBS containing 3% Triton X-100 (PBS-Triton). Washed, permeabilized tubules were immediately incubated overnight at 4°C with primary antibodies (Supplemental Table 1) diluted in PBS-Triton containing 5% BSA (Sigma-Aldrich). Labeled tubules were washed 7 times with PBS-Triton, and then incubated with corresponding fluorescent secondary antibodies (Supplemental Table 1) diluted in PBS-Triton for 1 hour at room temperature. Finally, tubules were washed 7 times with PBS-Triton, and then mounted with ProLong Gold Antifade Mountant (Thermo Fisher Scientific) and stained with DAPI. Confocal images were acquired (see “Kidney histology and immunofluorescence”).

### Generation of hiPSC-derived kidney organoids

Kidney organoids were generated following a previously established protocol (20, 42). The organoids were generated from hiPSCs obtained from 2 ADTKD-*UMOD* patients, and 1 unaffected patient described previously as “N2” (20). In brief, upon reaching a healthy 80%–90% confluence, 375,000 cells were seeded in a T25 flask in mTeSR1 media (STEMCELL Technologies) supplemented with 10 μM Rock inhibitor Y-27632 (Stem Cell technologies). After 12 hours, cells were treated with 8 μM CHIR 99021 (R&D Systems) in STEMdiff APEL2 Medium (STEMCELL Technologies) for 48 hours, followed by titration of CHIR 99021 to 10 μM for another 48 hours. Then, media were switched to STEMdiff APEL2 Medium with 200 ng/mL FGF9 (Preprotech) and 1 μg/mL heparin (Sigma-Aldrich) for the next 3 days. On day 8, cells were dissociated into single cells using Accutase (STEMCELL Technologies) for 5 minutes at 37°C. Five hundred thousand cells were pelleted at 400*g* for 2 minutes (twice, with a 180-degree flip after the first spin to increase the compactness of the pellet) and transferred on to a Transwell 0.4 μm pore polyester membrane (Corning, 3450). Pellets were then induced for 1 hour at 37°C with 5 μM CHIR 99021 in STEMdiff APEL2 Medium, and then kept with a maintenance media supplemented with 200 ng/mL FGF9 and 1 μg/mL heparin, as mentioned above until day 15. On day 16 and onwards, APEL2 media with 1 μg/mL heparin was changed every other day until day 26 of differentiation. On day 26, DMSO or 10 μM BRD4780 treatment was started and continued for 72 hours.

### Immunofluorescence of kidney organoids

After 72 hours of treatment, organoids were immersion fixed in PFA 4% for 20 minutes at room temperature, then washed twice with 1× PBS, and left overnight at 4°C in a 30% sucrose in PBS solution to dehydrate the tissue. The next day, the organoids were carefully mounted in cryomolds with OCT, snap frozen in dry ice and 100% ethanol, and stored at –80°C until further use. For immunofluorescence, 10 μm-thick sections were placed on Polylysine Adhesion Slides (Thermo Fisher Scientific), and either kept at –20°C for further use, or if used immediately, dried for 1 hour at room temperature, fixed with 4% PFA for 15 minutes, and permeabilized with 1% Triton X-100 in PBS. After fixation and permeabilization, sections were washed thoroughly with 0.1% Triton X-100/PBS and blocked for 1 hour at room temperature with 5% BSA in 1% Triton X-100/PBS. Primary antibodies were incubated at 4°C overnight in blocking solution. The next day, organoid sections were washed 3 times with 1% Triton X-100/PBS before incubating with secondary antibodies plus DAPI for 1 hour at room temperature. Last, sections were washed 3 times with 1% Triton X-100/PBS and prepared for imaging by mounting them with ProLong Gold Antifade Mountant. Confocal images were acquired (see “Kidney histology and immunofluorescence”).

### IP-MS sample processing

Samples were prepared in triplicate. Beads were washed twice with 50 mM Tris-HCl (200 μL, pH 7.5) and transferred to fresh 1.5 mL Eppendorf tubes and then further washed twice with 2 M urea/50 mM Tris-HCl (200 μL, pH 7.5). Proteins were digested with trypsin (5 μg/mL, 80 μL) in 2 M urea/50 mM Tris-HCl/1 mM dithiothreitol (DTT) at 25°C for 1 hour. Following a brief centrifugation step using a table-top centrifuge (5 to 10 seconds), supernatants were transferred to new 1.5 mL Eppendorf tubes. Beads were washed twice with 2 M urea/50 mM Tris-HCl (60 μL, pH 7.5), and supernatants were combined with respective supernatants from the first centrifugation step. Combined supernatants were centrifuged at 5,000*g* for 30 seconds to pellet remaining beads and the supernatants were transferred to new 1.5 mL Eppendorf tubes. Samples were reduced with DTT (4 mM, on shaker at 1,000 rpm, 30 minutes, 25°C) and alkylated with iodoacetamide (10 mM) for 45 minutes at 25°C in the dark. Proteins were digested overnight with trypsin (0.5 μg/μL in trypsin buffer, 1:100 [w/w] enzyme/protein ratio, 25°C, on shaker at 700 rpm). Samples were acidified with formic acid (1% FA, 200 μL, pH < 3) and peptides were desalted using C18 stage tips following a standard protocol (58). Briefly, stage tips were activated with 50% acetonitrile (ACN), 0.1% FA (50 μL, 1,500*g*) and conditioned with 0.1% FA (50 μL, 1,500*g*, twice). Samples (350 μL) were loaded on the tips and spun at 1,500*g* until all volume flowed through completely without drying the stage tips. Samples were washed with 0.1% FA (50 μL, twice, 1,500*g*), eluted with 50% ACN/0.1% FA (50 μL, 1,500*g*), and lyophilized. Peptides were subsequently reconstituted in fresh HEPES (50 mM, 95.3 μL, pH 7.5) for tandem mass tag (TMT) labeling. Total peptide quantities were quantified using an EvoSep One LC system coupled to a Q-Exactive Plus MS system (Thermo Fisher Scientific). A calibration curve of Jurkat cell digestion was generated with the following total protein amounts: 31.3 ng, 62.5 ng, 125 ng, 250 ng, 500 ng, and 1 μg. The samples were analyzed with the same system. The total area under the total ion current trace was used to estimate the total peptide amount in each sample. Samples were then labeled with TMTs as follows: empty vector (126, 127N, 127C), UMOD^C126R^ (128N, 128C, 129C), and UMOD^WT^ (130N, 130C, 131). TMT labeling occurred for 1 hour at room temperature with shaking (800 rpm), following standard protocol. Samples were quenched with 5% hydroxylamine (8 μL, 20°C, 700 rpm). The TMT labeling reaction efficiency was checked using the EvoSep System coupled to a Q-Exactive Plus MS system. One microliter was injected into the system and the percentage of fully labeled peptides was greater than 95%. After checking the TMT labeling efficiency, all channels were combined in 1 vial and lyophilized. The combined samples were reconstituted in 3% ACN/5% FA and fractionated using high-pH reverse-phase chromatography with C18 disks. Briefly, 3 C18 discs were placed in 200 μL pipette tips. The tips were conditioned with methanol (100 μL, 2,200*g* 3 minutes), followed by 100 μL of 50% ACN/1% FA (100 μL, 2,200*g*, 1 minute). Tips were equilibrated with 0.1% trifluoroacetic acid (100 μL, 2,200*g*, 3.5 minutes) prior to sample loading (100 μL, 2,200*g*, 3 minutes). The sample was washed twice with 1% FA (100 μL, 2,200*g*, 3.5 minutes, twice). A stepwise elution occurred using 20 mM ammonium formate/3% ACN (pH 9, 250 μL, 2,500*g*, 6 minutes, fraction 0), 8% ACN in 20 mM ammonium formate (pH 9, 300 μL, 2,500*g*, 7 minutes, fraction 1), 12% ACN in 20 mM ammonium formate (pH 9, 300 μL, 2,500*g*, 7 minutes, fraction 2), 16% ACN in 20 mM ammonium formate (pH 9, 300 μL, 2,500*g*, 7 minutes, fraction 3), 20% ACN in 20 mM ammonium formate (pH 9, 300 μL, 2,500*g*, 7 minutes, fraction 4), 25% ACN in 20 mM ammonium formate (pH 8.5, 300 μL, 2,500*g*, 7 minutes, fraction 5), and 50% ACN in 20 mM ammonium formate (pH 8.5, 300 μL, 2,500*g*, 7 minutes, fraction 6). Samples were transferred to HPLC vials, lyophilized, and resuspended in 3% ACN/5% FA (8 μL) for nano–LC-MS/MS analysis.

### MS analysis

Fractionated samples were analyzed on an Orbitrap Q-Exactive HF Plus MS (Thermo Fisher Scientific) equipped with a nanoflow ionization source and coupled to a nanoflow Proxeon EASY-nLC 1000 UHPLC system (Thermo Fisher Scientific). Acquisition occurred in positive-ion mode. Samples were injected on an in-house packed column (22 cm × 75 μm diameter C18 silica picofrit capillary column) heated at 50°C. The mobile phase flow rate was 250 nL/min of 3% ACN/1% FA (solvent A) and 90% ACN/ 0.1% FA (solvent B). Peptides were separated using the following LC gradient: 0%–6% B in 1 minute, 6%–30% B in 85 minutes, 30%–60% B in 9 minutes, 60%–90% B in 1 minute, maintain 90% B for 5 minutes, 90%–50% B in 1 minute, and maintain 50% B for 5 minutes. Data were acquired in profile and centroid modes for the MS1 and MS2 scans, respectively. Samples were analyzed in data-dependent analysis (DDA) mode using a Top-20 method. Ion source parameters were spray voltage of 2 kV and source temperature of 250°C. Full MS scans were acquired in the *m*/*z* range 350–1,800, with an automatic gain control (AGC) target of 3 × 10^6^, maximum injection time (IT) of 10 ms, and resolution of 60,000 (at *m*/*z* 200). MS/MS parameters were as follows: AGC target 5 × 10^4^, maximum IT 105 ms, loop count 20, isolation window 0.7 *m*/*z*, isolation offset 0.0 *m*/*z*, normalized collision energy (NCE) 31, resolution 45,000 (at *m*/*z* 200), and fixed first mass 100 *m*/*z*; unassigned, 1, 7, 8, and more than 8 charged ions were excluded from MS/MS. Dynamic exclusion was set to 15 seconds.

### Proteomic data analysis

Raw MS data were analyzed using Spectrum Mill Proteomics Workbench (prerelease version B.06.01.202, Agilent Technologies). A trypsin-specific enzyme search was performed against the 2017 UniProt human FASTA file (UniProt.human.20171228.RISnrNF.553smORFs.264contams) containing 65,095 entries. Peptide and fragment tolerances were at 20 ppm, minimum matched peak intensity 40%, and peptide FDRs were fixed at 1%. Fixed modifications were cysteine carbamidomethylation and TMT 10 (N-term, K), and variable modifications were Acetyl (protein N-term), Oxidized methionines (M), Pyroglutamic acid (N-term Q), and Deamidation (N). The precursor ion mass shift range was set to –18 and 70 Da. Spectra with a score of less than 4 were filtered out. Peptides were validated using the following parameters: for charge states 2–4, an FDR of 1.2 was applied to each run and for charge state 5, an FDR of 0.6 was applied across all runs. Results were further validated at the protein level and proteins with a score of 20 or higher were accepted as valid. Reporter ion correction factors, specific to the TMT batch (batch number TE270748), were applied. A protein/peptide summary was generated using the median across all TMT channels as the denominator. Shared peptides were assigned to the protein with the highest score using the protein grouping method “expand subgroups, top uses shared SubGroup Top (SGT)”.

### Bioinformatics analysis for IP-MS

Calculated ratios at the protein level were imported into Protigy v0.8.9.3 for normalization and features selection (https://github.com/broadinstitute/protigy). To account for variability between samples, log ratios were normalized by centering using the sample Median and scaled using the sample Median Absolute Deviation (Median MAD). A 2-sample moderated *t* test was performed to identify proteins whose expression changed significantly across conditions. A linear model using one contrast (Mutant – WT) to correct for the negative control (empty vector) in each case by calculating the difference between these 2 conditions (Linear Model App version 1). Gene Ontology (GO) overrepresentation analysis of proteins in the resulting clusters was performed with the gProfiler R package (https://cran.r-project.org/web/packages/gprofiler2/index.html).

### Statistics

All statistical analyses and graphical representations were produced with GraphPad Prism version 10. Specific analyses are described in the corresponding figure legends. Normality tests guided the selection of appropriate statistical tests for subsequent data analysis.

### Study approval

#### Mouse model.

All procedures were IACUC approved under Broad Institute animal protocol no. 0061-07-15-1.

#### Human cell lines.

The cells recovered from patients were obtained following IRB approval under the protocol designated IRB00000352 at Wake Forest University Health Sciences, titled “Characterization of Individuals with Inherited Kidney Disease Sub-Study: Induced Pluripotent Stem Cell Analysis.” We ensured that all cellular materials were procured with full compliance to ethical guidelines and patient consent.

### Data availability

All data presented in this manuscript and materials related to this study can be provided upon request. Numerical values corresponding to each data point depicted in the graphs are documented in the Supporting Data Values file. The original mass spectra and the protein sequence database used for searches has been deposited in the public proteomics repository MassIVE (http://massive.ucsd.edu) and are accessible at ftp://MSV000093889@massive.ucsd.edu when providing the dataset password: UMOD. If requested, also provide the username: MSV000093889.

## Author contributions

SBV, SLA, JLBP, and AG developed and wrote the manuscript. SBV, JLBP, MKA, and AG conceptualized and planned experiments. SBV, JZ, MRK, EG, EHS, KHK, CQ, HD, SAJV, and NH performed experiments. MR, JL, and ACG provided technical support during experiments. SBV, MRB, JZ, MRK, EHS, SS, HD, and MKA performed data analysis. SK, MŽ, KK, and AJB provided essential cell lines and patient samples. SBV, MDL, MP, NU, SAC, NH, MB, KM, SB, JH, AW, MKA, JLBP, JLS, and AG provided oversight and/or mentorship.

## Supplementary Material

Supplemental data

Unedited blot and gel images

Supporting data values

## Version 1 12/16/2024

Electronic publication

## Version 2 12/18/2024

Corrected figure 5
