# Supplementary material for: Disrupted uromodulin trafficking is rescued by targeting TMED cargo receptors
Source: J Clin Invest. 2024 Dec 16;134(24):e180347. doi: 10.1172/JCI180347 (PMC11645142; doi:10.1172/JCI180347)
Supplement: Supplemental data [file jci-134-180347-s008.pdf]

Supplementary Table 1. List of antibodies.

Antibody	Source	Identifier
Tissue IF and Western blot		
Sheep polyclonal anti-Uromodulin	Meridian Life Sciences	Cat#: K90071C; RRID:AB_153128
Mouse monoclonal anti-TMED2 (E-12)	Santa Cruz Biotechnology	Cat#: sc-376458; RRID:AB_11151031
Rabbit polyclonal anti-TMED10	Bethyl Laboratories	Cat#: A305-219A; RRID:AB_2631612
Rabbit polyclonal anti-TMED9	Proteintech	Cat#: 21620-1-AP; RRID: AB_10858623
Mouse monoclonal anti-TMED10	Santa Cruz Biotechnology	Cat# sc-376459; RRID:AB_11150297
Rabbit polyclonal anti-Calnexin	Abcam	Cat#: ab22595; RRID: AB_2069006
Rabbit polyclonal anti-CD45	Abcam	Cat#: ab10558; RRID: AB_442810
Armenian monoclonal hamster anti-MUC1	Abcam	Cat#: ab80952; RRID: AB_1640314
Fluorescein labeled Lotus Tetragonolobus Lectin (LTL)	Vector Laboratories	Cat#: FL-1321; RRID: AB_2336559
Rabbit polyclonal anti-C125RUMOD	Innovagen	The antibody was raised against the mouse UMOD sequence (CHALATRVNTEGD)
Mouse monoclonal anti α-sma	Sigma-Aldrich	Cat# A5228, RRID:AB_262054
Guinea pig polyclonal anti-NKCC2	In-house developed by the Bachmann Lab	The antibody was raised against 85 NH2 -terminal amino acids of NKCC2 (for more information please refer to Schmitt R et al. 2003 (59))
Rabbit monoclonal anti-XBP1-s (D2C1F)	Cell Signaling Technology	Cat#: 12782; RRID:AB_2687943
Rabbit monoclonal anti-Vinculin (E1E9V) XP	Cell Signaling Technology	Cat#: 18799; RRID:AB_2728768
Rabbit polyclonal anti α/β-tubulin	Cell Signaling Technology	Cat#: 2148S; RRID:AB_2288042

Western blot in cells		
Mouse monoclonal anti-FLAG	Sigma-Aldrich	Cat# F3165; RRID:AB_259529
Mouse monoclonal anti-GFP (4B10)	Cell Signaling Technology	Cat# 2955; RRID:AB_1196614
Mouse monoclonal anti-V5	Thermo Fisher Scientific	Cat# R961-25; RRID:AB_2556565
Mouse monoclonal anti-Myc	Santa Cruz Biotechnology	Cat# sc-40; RRID:AB_627268
4i		
Rabbit polyclonal anti-TMED9	Proteintech	Cat#: 21620-1-AP; RRID: AB_10858623
Rabbit polyclonal anti-ERGIC-53	Proteintech	Cat# 13364-1-AP; RRID:AB_2135994
Mouse monoclonal anti-GM130	BD Biosciences	Cat# 610822; RRID:AB_398141
Mouse monoclonal anti-Sec13 (F-6)	Santa Cruz Biotechnology	sc-514308, RRID:AB_2186242
Rabbit polyclonal anti-COPB2	Thermo Fisher Scientific	Cat# PA5-96557; RRID:AB_2808359
Mouse monoclonal anti-Rab7	Cell Signaling Technology	Cat# 95746; RRID:AB_2800252
Rabbit monoclonal anti-EEA1 (C45B10)	Cell Signaling Technology	Cat# 3288; RRID:AB_2096811
Mouse monoclonal anti-TGN38	Novus Biologicals	Cat# NB300-575; RRID:AB_2240559
Rabbit polyclonal anti-Calnexin	Abcam	Cat# ab22595; RRID:AB_2069006
Secondaries		
Goat anti-Rabbit IgG (H+L) Cross-Absorbed, Alexa Fluor 488	Thermo Fisher Scientific	Cat# A-11008; RRID:AB_143165

Goat anti-Mouse IgG (H+L) Cross-Absorbed, Alexa Fluor 488	Thermo Fisher Scientific	Cat# A-11001; RRID:AB_2534069
Donkey anti-Sheep IgG (H+L) Cross-Absorbed, Alexa Fluor 568	Thermo Fisher Scientific	Cat# A-21099; RRID:AB_10055702
Goat anti-Rabbit IgG (H+L) Cross-Absorbed, Alexa Fluor 568	Thermo Fisher Scientific	Cat# A-11011; RRID:AB_143157
Goat anti-Mouse IgG (H+L) Cross-Absorbed, Alexa Fluor 568	Thermo Fisher Scientific	Cat# A-11004; RRID:AB_2534072
Goat anti-Armenian Hamster IgG (H+L) Highly Cross-Adsorbed Secondary Antibody, Alexa Fluor 647	Thermo Fisher Scientific	Cat# A78967, RRID:AB_2925790
Goat anti-Guinea Pig IgG (H+L) Highly Cross-Adsorbed Secondary Antibody, Alexa Fluor 647	Thermo Fisher Scientific	Catalog # A-21450, RRID:AB_141882
Donkey, polyclonal Cy3-coupled donkey anti-mouse IgG	Dianova	Cat# 715-165-150, RRID:AB_2340813

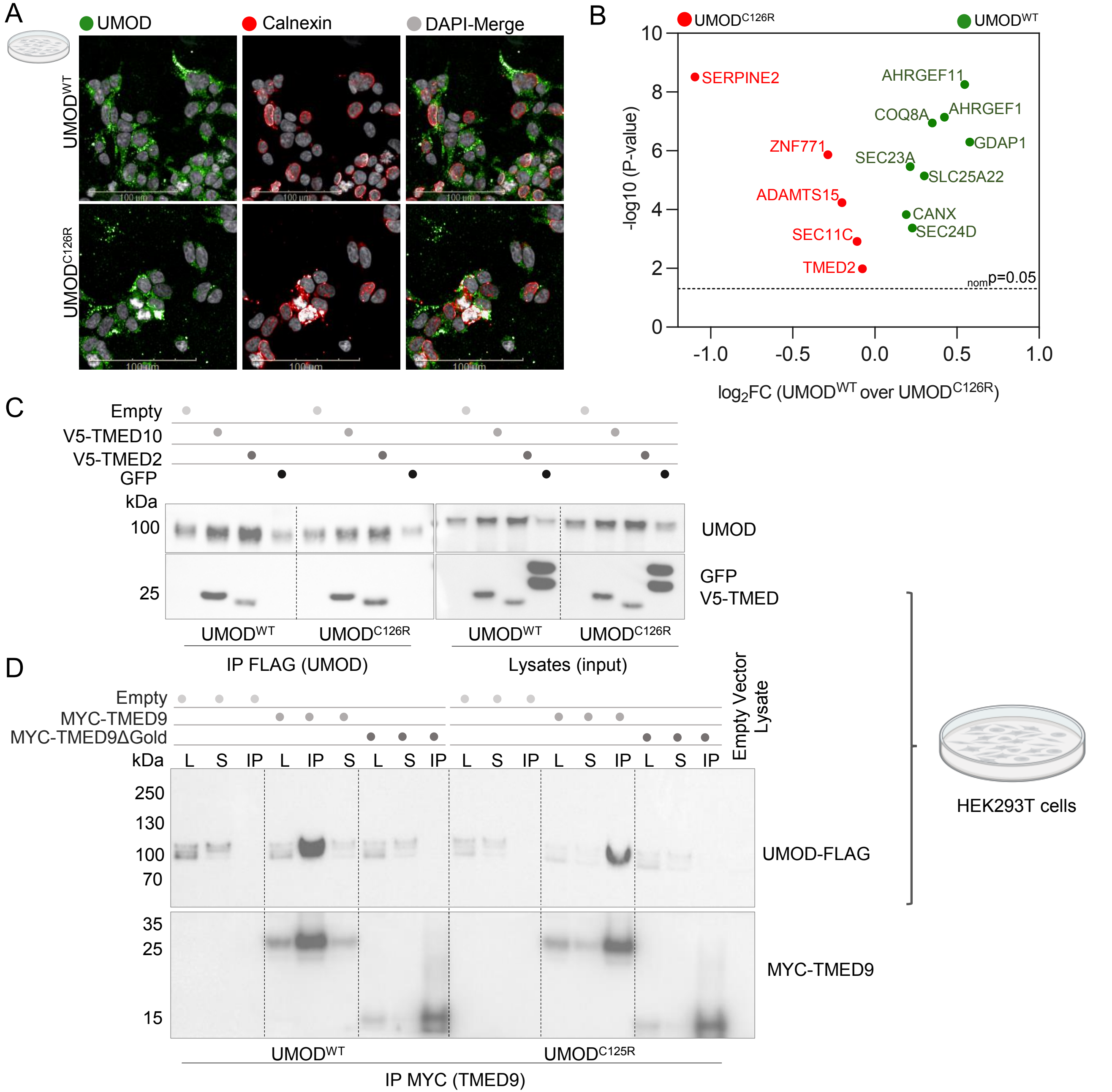
A
UMOD
Calnexin
DAPI-Merge
UMODWT
UMODC126R
100 µm
B
UMODC126R
UMODWT
-log10 (P-value)
log2FC (UMODWT over UMODC126R)
SERPINE2
ZNF771
ADAMTS15
SEC11C
TMED2
AHRGEF11
COQ8A
AHRGEF1
GDAP1
SEC23A
SLC25A22
CANX
SEC24D
nomp=0.05
C
Empty
V5-TMED10
V5-TMED2
GFP
kDa
100
25
UMOD
GFP
V5-TMED
UMODWT
UMODC126R
IP FLAG (UMOD)
Lysates (input)
D
Empty
MYC-TMED9
MYC-TMED9ΔGold
kDa
L S IP L IP S L S IP L S IP L S IP L S IP
250
130
100
70
35
25
15
UMOD-FLAG
MYC-TMED9
Empty Vector Lysate
UMODWT
UMODC125R
IP MYC (TMED9)
HEK293T cells

Supplementary Figure 1. Uromodulin binds the TMED family of cargo receptors. A. Immunofluorescence images of HEK293T cells transiently transfected with wild-type UMOD (UMODWT) or mutant misfolded UMOD (UMODC126R), fixed and stained for UMOD (green), DAPI (gray), and calnexin (red) to corroborate ER retention. Scale bar = 100 µm **B.** Volcano plots depicting IP-MS of flag-tagged-UMOD wild-type (UMODWT) or mutant misfolded UMOD (UMODC126R) from HEK293T cells. The x-axis shows the log2 fold-change of protein enrichment in the wild-type protein (WT) pulldown over the mutant (C126R) pulldown. Significant interactors are highlighted in red and green. The right hand-side of the volcano plot depicts the proteins (green) that preferentially bind to wild-type UMOD. The left-hand side of the volcano plot depicts the proteins (red) that preferentially bind to mutant-misfolded UMOD. n=3 technical replicates. **C.** Co-immunoprecipitation of FLAG from HEK293T cells co-transfected with wildtype UMOD (UMODWT) or mutant UMOD (UMODC125R), both FLAG-tagged, and TMED10 or TMED2 (both V5-tagged). An additional group was co-transfected with UMOD and GFP to show specificity of TMED binding. Left, immunoprecipitation. Right, whole-cell lysate that served as input. **D.** Co-immunoprecipitation of Myc from HEK293T cells co-transfected with wild-type UMOD (UMODWT) or mutant UMOD (UMODC125R), both FLAG-tagged, and Myc-tagged TMED9 or Myc-TMEDΔGOLD, a TMED9 mutant lacking the cargo-binding GOLD domain. L=Lysate, S=Supernatant, IP=immunoprecipitation.

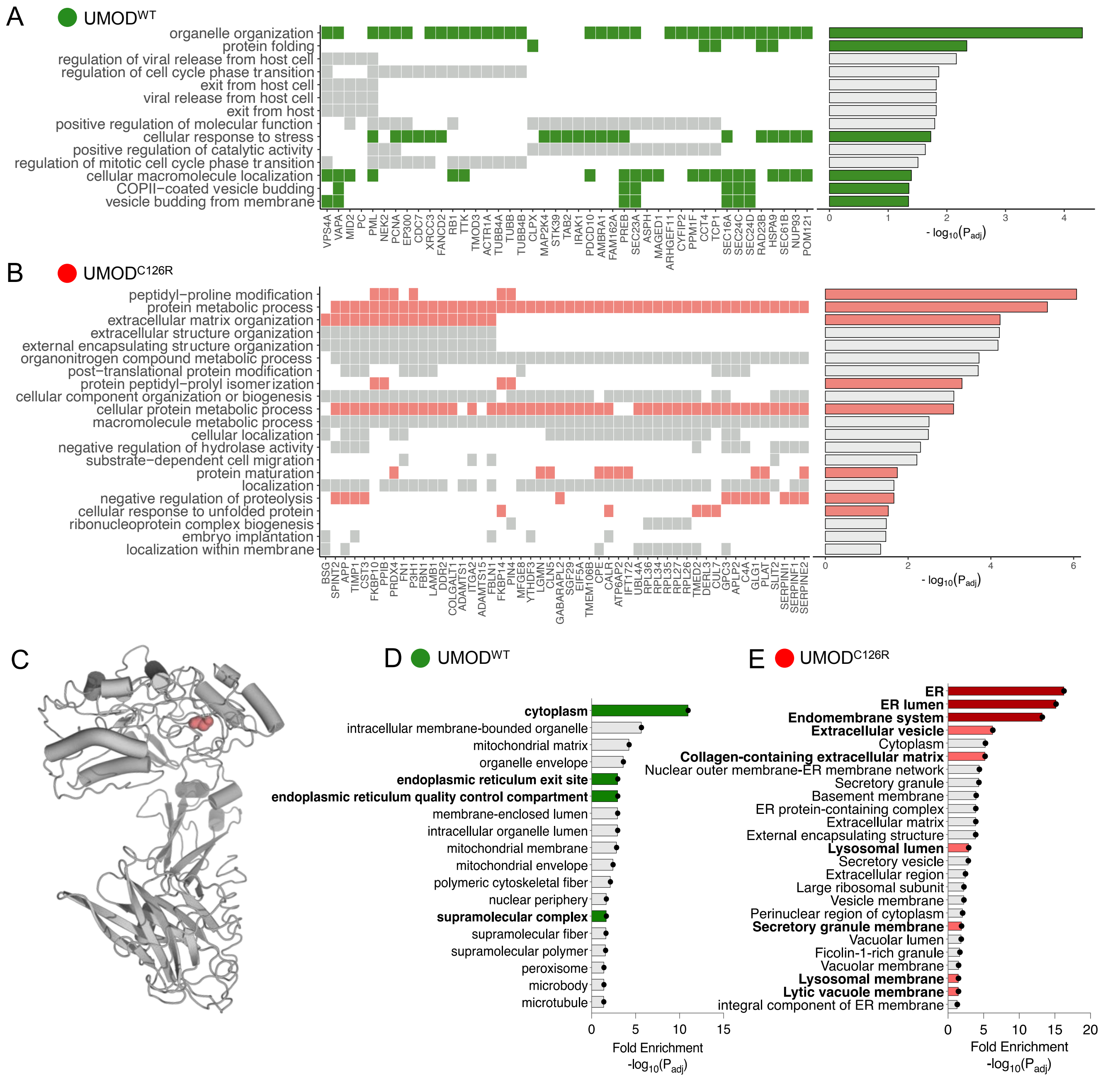

A
UMODWT
organelle organization
protein folding
regulation of viral release from host cell
regulation of cell cycle phase transition
exit from host cell
viral release from host cell
exit from host
positive regulation of molecular function
cellular response to stress
positive regulation of catalytic activity
regulation of mitotic cell cycle phase transition
cellular macromolecule localization
COPII−coated vesicle budding
vesicle budding from membrane
VPS4A VAPA MID2 PC PML NEK2 PCNA EP300 CDC7 XRCC3 FANCD2 RB1 TTK TMOD3 ACTR1A TUBB4A TUBB TUBB4B CLPX MAP2K4 STK39 TAB2 IRAK1 PDCD10 AMBRA1 FAM162A PREB SEC23A ASPH MAGED1 ARHGEF11 CYFIP2 PPM1F CCT4 TCP1 SEC16A SEC24C SEC24D RAD23B HSPA9 SEC61B NUP93 POM121
0 1 2 3 4
- log10(Padj)
B
UMODC126R
peptidyl−proline modification
protein metabolic process
extracellular matrix organization
extracellular structure organization
external encapsulating structure organization
organonitrogen compound metabolic process
post−translational protein modification
protein peptidyl−prolyl isomerization
cellular component organization or biogenesis
cellular protein metabolic process
macromolecule metabolic process
cellular localization
negative regulation of hydrolase activity
substrate−dependent cell migration
protein maturation
localization
negative regulation of proteolysis
cellular response to unfolded protein
ribonucleoprotein complex biogenesis
embryo implantation
localization within membrane
BSG SPINT2 APP TIMP1 CST3 FKBP10 PPIB PRDX4 FN1 P3H1 FBN1 LAMB1 DDR2 COLGALT1 ADAMTS1 ITGA2 ADAMTS15 FBLN1 FKBP14 PIN4 MFGE8 YTHDF3 LGMN CLN5 GABARAPL2 SGF29 EIF5A TMEM106B CPE CALR ATP6AP2 IFT172 UBL4A RPL36 RPL34 RPL35 RPL27 RPL26 TMED2 DERL3 CUL7 GPC3 APLP2 C4A GLG1 PLAT SLIT2 SERPINI1 SERPINF1 SERPINE2
0 2 4 6
- log10(Padj)
C
D
UMODWT
cytoplasm
intracellular membrane-bounded organelle
mitochondrial matrix
organelle envelope
endoplasmic reticulum exit site
endoplasmic reticulum quality control compartment
membrane-enclosed lumen
intracellular organelle lumen
mitochondrial membrane
mitochondrial envelope
polymeric cytoskeletal fiber
nuclear periphery
supramolecular complex
supramolecular fiber
supramolecular polymer
peroxisome
microbody
microtubule
0 5 10 15
Fold Enrichment
-log10(Padj)
E
UMODC126R
ER
ER lumen
Endomembrane system
Extracellular vesicle
Cytoplasm
Collagen-containing extracellular matrix
Nuclear outer membrane-ER membrane network
Secretory granule
Basement membrane
ER protein-containing complex
Extracellular matrix
External encapsulating structure
Lysosomal lumen
Secretory vesicle
Extracellular region
Large ribosomal subunit
Vesicle membrane
Perinuclear region of cytoplasm
Secretory granule membrane
Vacuolar lumen
Ficolin-1-rich granule
Vacuolar membrane
Lysosomal membrane
Lytic vacuole membrane
integral component of ER membrane
0 5 10 15 20
Fold Enrichment
-log10(Padj)

Supplementary Figure 2. The Uromodulin interactome. Functional annotation of proteins significantly enriched in UMOD's interactome. Gene Ontology (GO) enrichment analysis was performed in the web tool gProfiler with the list of genes significantly enriched ($p \leq 0.05$). GO representing Biological Pathways (**A. and B.**) and Cell Component (**D. and E.**) are presented in different graphs. Redundant terms were removed from graphs. Relevant terms are highlighted in Bold Font. **C.** TSSER UMOD structure based on 4wrn, modeled in PyMol. The location of C126 (the cysteine mutation that we studied in this project) is indicated with red spheres. The predicted structure of uromodulin contains four EGF-like domains, a cysteine-rich D8C domain (far left), and a bipartite Zona Pellucida domain (far right). All domains were gray-shaded for simplicity. Mutation C126R is in the EGF-like domain III.

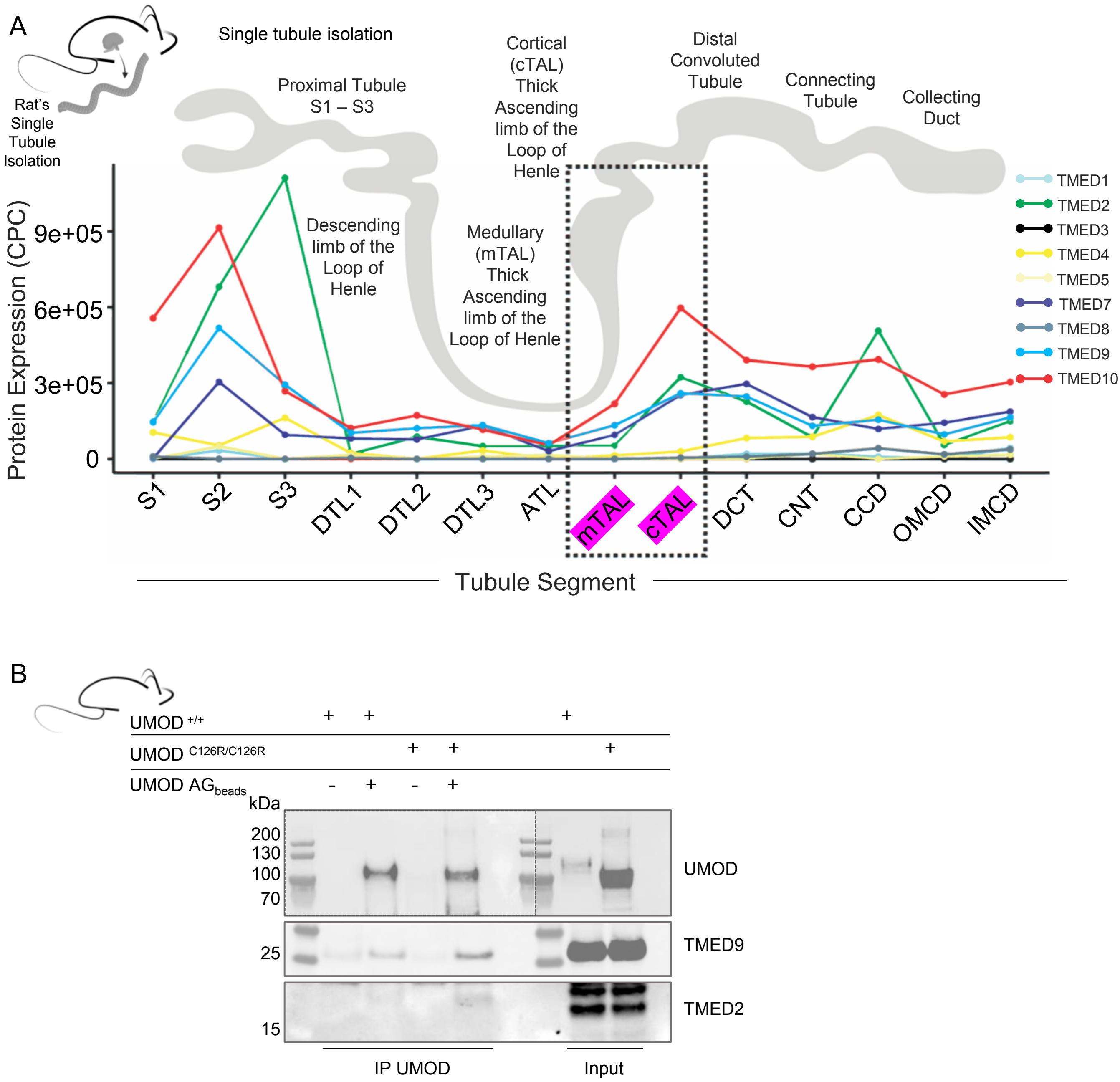

A
Single tubule isolation
Rat's Single Tubule Isolation
Proximal Tubule S1 – S3
Cortical (cTAL) Thick Ascending limb of the Loop of Henle
Distal Convoluted Tubule
Connecting Tubule
Collecting Duct
Descending limb of the Loop of Henle
Medullary (mTAL) Thick Ascending limb of the Loop of Henle
Protein Expression (CPC)
9e+05
6e+05
3e+05
0
TMED1
TMED2
TMED3
TMED4
TMED5
TMED7
TMED8
TMED9
TMED10
S1
S2
S3
DTL1
DTL2
DTL3
ATL
mTAL
cTAL
DCT
CNT
CCD
OMCD
IMCD
Tubule Segment
B
UMOD +/+
UMOD C126R/C126R
UMOD AGbeads
kDa
200
130
100
70
25
15
UMOD
TMED9
TMED2
IP UMOD
Input

Supplementary Figure 3. TMED expression along the nephron and relevant in vivo interaction between TMEDs and UMOD. **A.** Relative protein expression levels of the TMEDs in renal tubule segments. Data analyzed from Limbutara et al. JASN 2020 (available at ProteomeXchange Consortium (http://proteomecentral. proteomexchange.org)). **B.** Co-immunoprecipitation of UMOD from whole-kidney extract of wild-type ($UMOD^{+/+}$) or homozygous mutant ($UMOD^{C125R/C125R}$) mice. We used agarose beads bound to a mouse monoclonal antibody that recognizes UMOD (UMOD AG beads) or agarose beads bound to control mouse IgG isotype. At left, immunoprecipitation (IP UMOD). At right, whole-kidney lysate that served as the input.

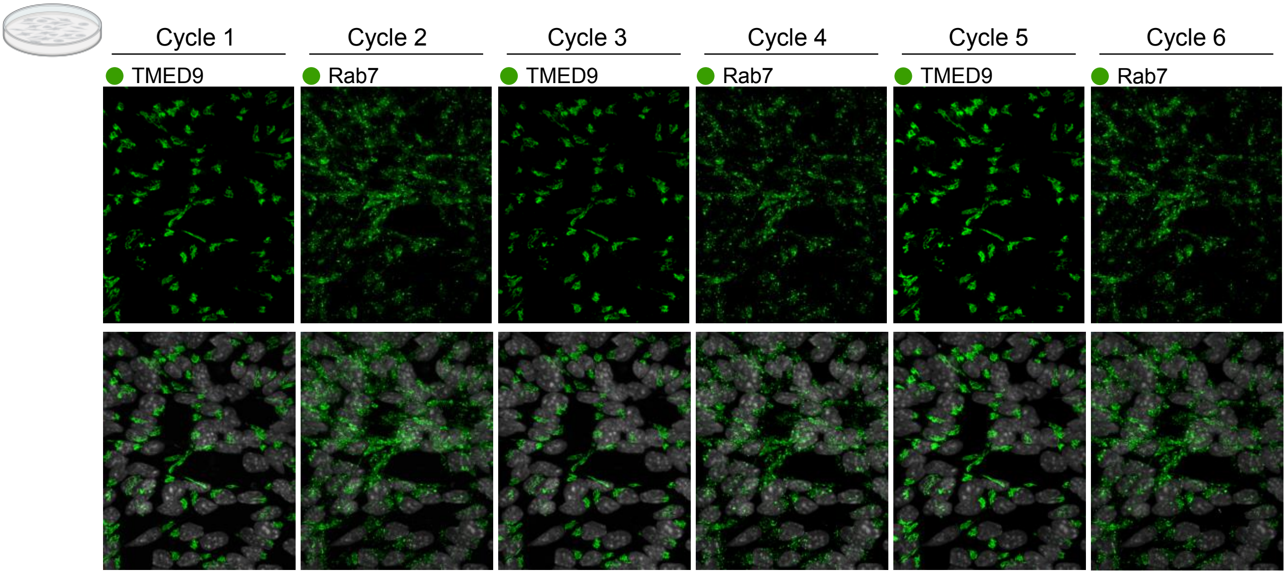

Supplementary Figure 4. Iterative Indirect Immunofluorescence Imaging (4i) of Uromodulin in epithelial cells. Representative immunofluorescence images depicting the efficacy of antibody elution and re-staining across successive imaging cycles in the same cells. The green depicts either TMED9 or Rab7, depending on the cycle.

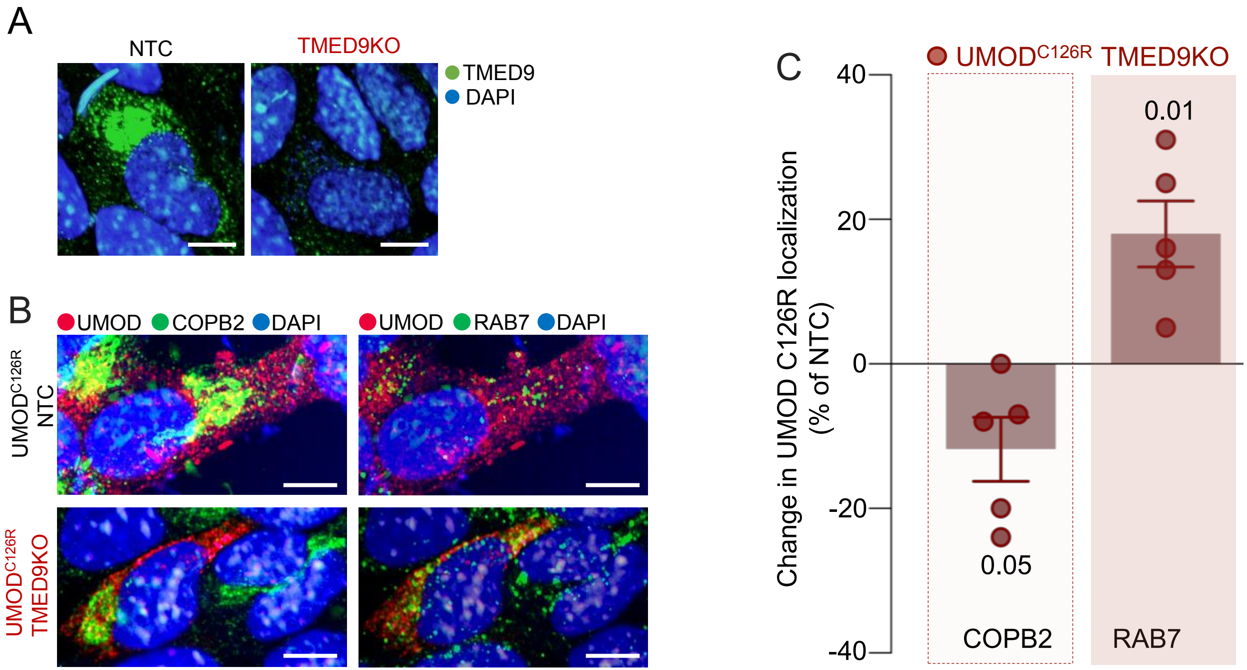
A
NTC
TMED9KO
TMED9
DAPI
B
UMOD
COPB2
DAPI
UMOD
RAB7
DAPI
UMODC126R NTC
UMODC126R TMED9KO
C
UMODC126R TMED9KO
Change in UMOD C126R localization (% of NTC)
40
20
0
-20
-40
0.01
0.05
COPB2
RAB7

Supplementary Figure 5. TMED9 deletion recapitulates BRD4780-induced forward trafficking of mutant UMOD. A. Immunofluorescence images of AtT20 stably transfected with $UMOD^{C126R}$ with/without CRISPR/Cas9 stable TMED9 deletion. NTC=Non-Targeting Control guide RNA, TMED9KO=TMED9-targeting guide RNA. Scale bar = 25 µm. **B.** Immunofluorescence images of AtT20 cells stably transfected with mutant misfolded UMOD ($UMOD^{C126R}$) in either NTC or TMED9KO conditions. 4i was performed as described in the Methods. Scale bar = 25 µm. **C.** Quantification of B. represented as change induced by TMED9KO from a baseline of the cells transfected with NTC. n=6 technical replicates. One sample t-test against a theoretical mean of 0. Each data point represents the mean values per well of all the cells in a well. Mean±SEM.

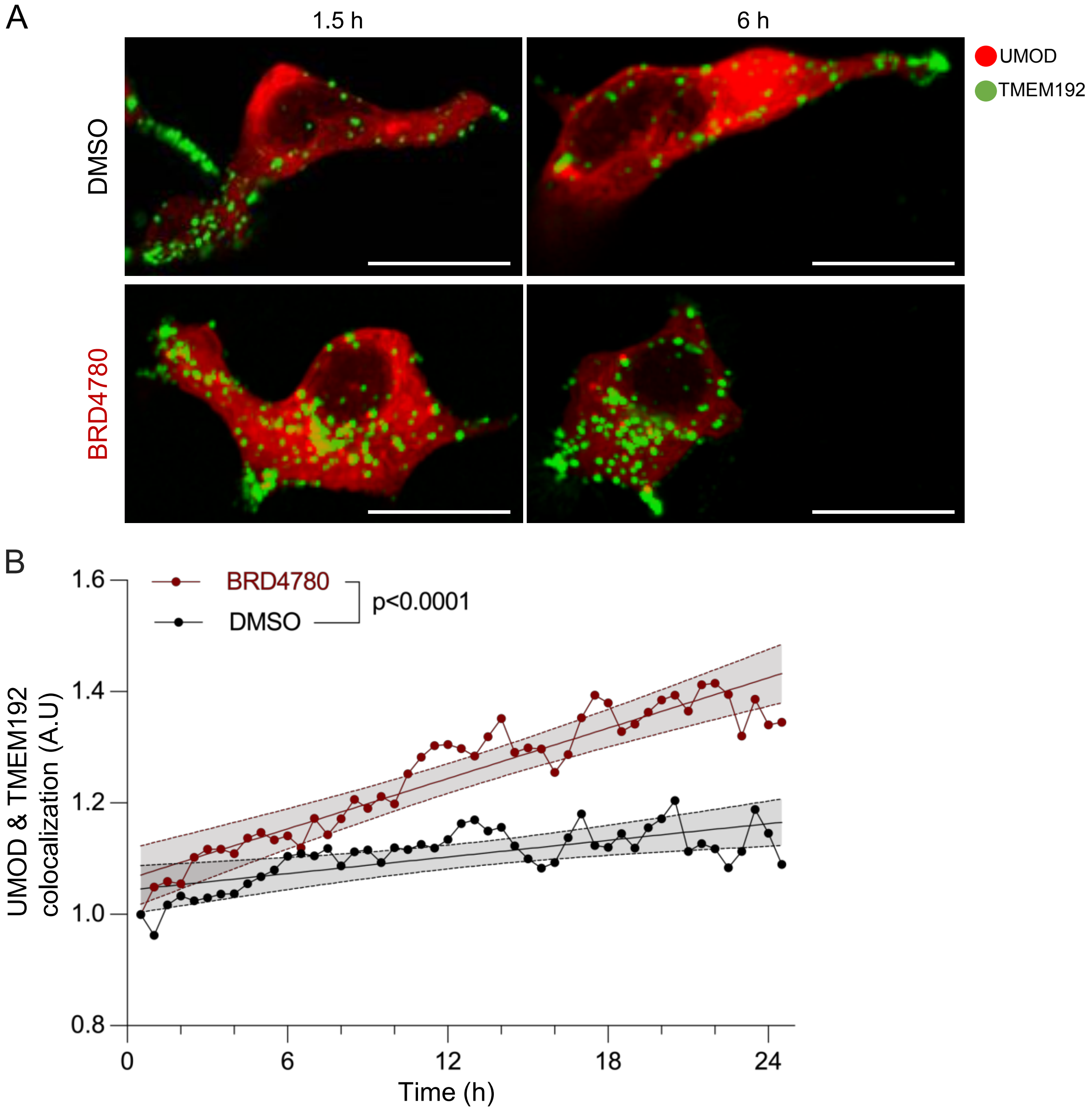
A
1.5 h
6 h
UMOD
TMEM192
DMSO
BRD4780
B
BRD4780
DMSO
p<0.0001
UMOD & TMEM192 colocalization (A.U)
1.6
1.4
1.2
1.0
0.8
0
6
12
18
24
Time (h)

Supplementary Figure 6. BRD treatment promotes colocalization of UMOD and lysosomal markers over time. A. Immunofluorescence images depicting the subcellular localization dynamics of mutant UMOD (UMODC126R) fused to the mScarlet fluorescent protein (UMODC126R-mScarlet) and a construct encoding the lysosomal protein TMEM192 tagged with GFP (TMEM192-GFP). HEK293T cells were treated for 24-hours with either DMSO or 5 μM BRD4780. Scale bar = 10 μm. **B.** Quantification of overlap between UMODC126R-mScarlet- and TMEM192-GFP-positive areas. Linear regression analysis shows significantly different (p-value<0.0001) slopes of straight interpolation lines between both treatment groups, demonstrating the efficacy of BRD4780 to increase colocalization of UMOD and TMEM192. Shown are aggregate data from two biological replicates. 95% confidence intervals are indicated by colored bands, black for DMSO, red for BRD4780. Data points represent the Mean±SEM of two independent experiments, each experiment has 3 technical replicates (n=6 wells per condition).

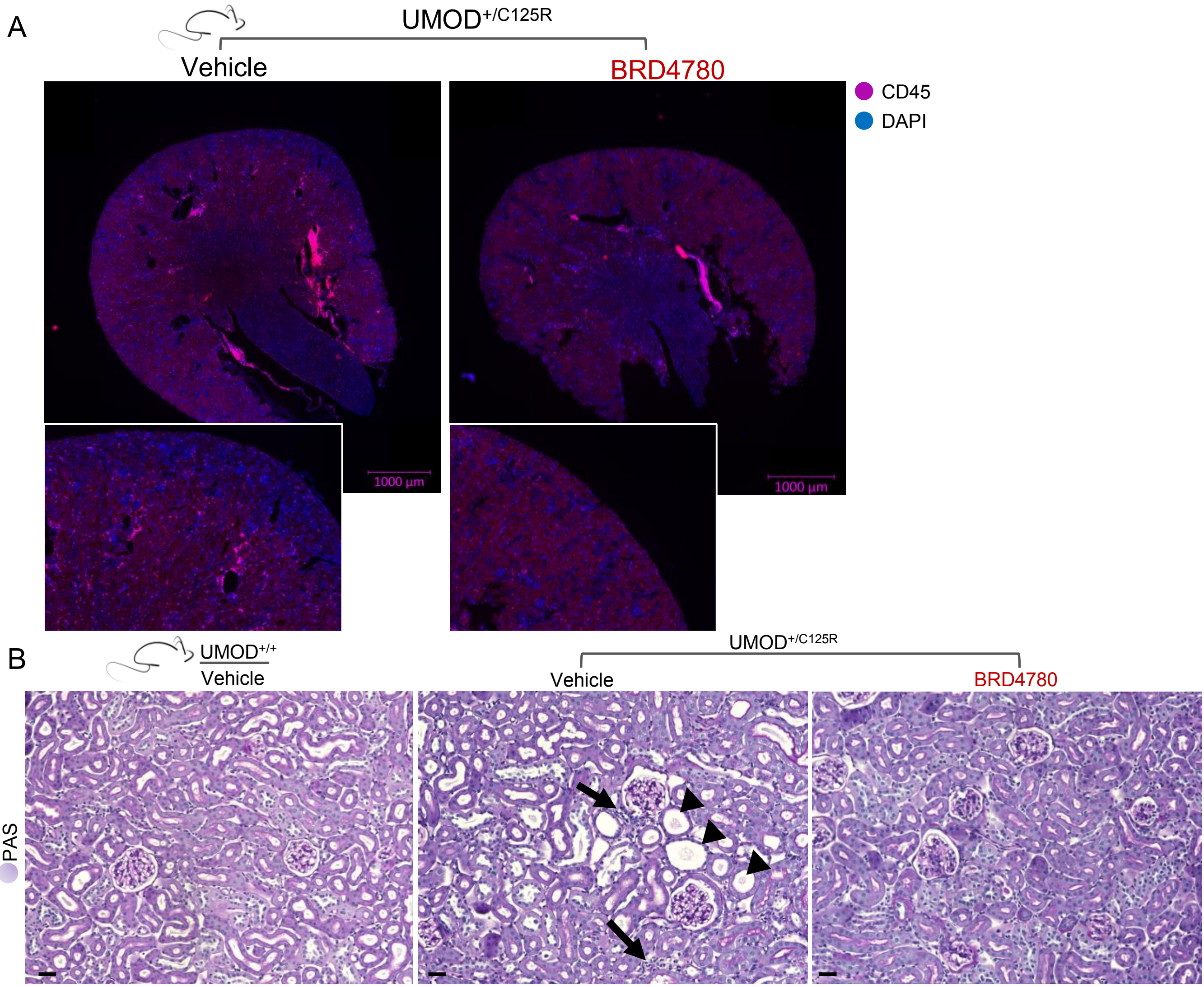
A
UMOD+/C125R
Vehicle
BRD4780
CD45
DAPI
1000 µm
1000 µm
B
UMOD+/+
Vehicle
UMOD+/C125R
Vehicle
BRD4780
PAS

Supplementary Figure 7. Assessment of leukocyte Infiltration via CD45 Staining and histological alterations via PAS Staining. A. Low magnification (10x) images of whole kidney sections from the vehicle-treated mouse stained with CD45, revealing extensive CD45+ leukocyte infiltration. In contrast, images of whole kidney sections from the treated group revealed a marked reduction in CD45+ leukocyte infiltration post-treatment. **B.** PAS staining of paraffin-embedded kidney sections. Black arrows indicate interstitial inflammatory infiltration, arrowheads indicate dilated TALs. Scale bar = 40 µm.

Graphical abstract

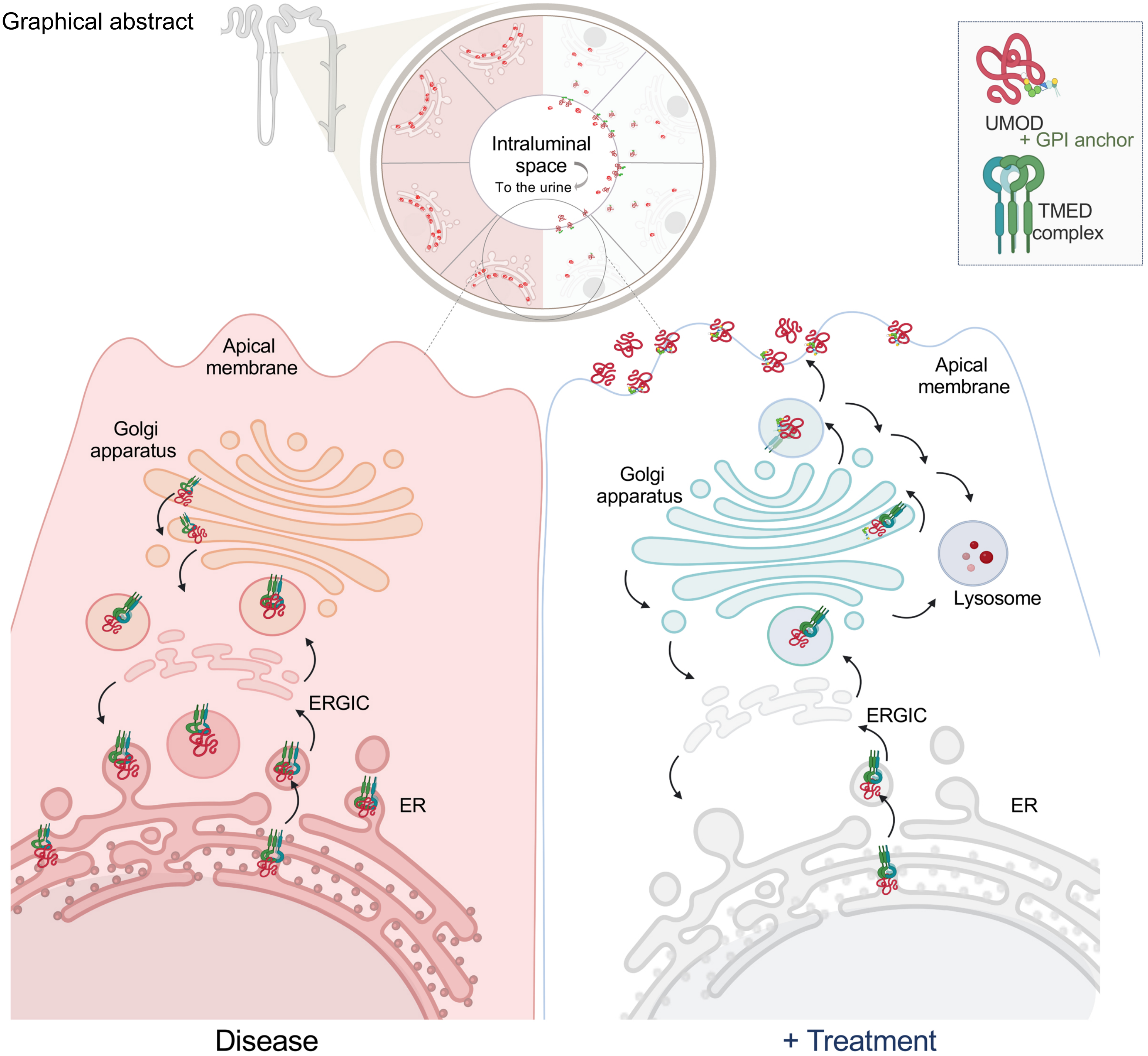

Targeting TMEDs corrects UMOD trafficking, alleviates ER stress, and mitigates other pathological effects. Schematic depicts isolated tubules: on the left, vehicle-treated mice display intracellular UMOD accumulation and expanded ER. On the right, BRD4780-treated mice exhibit UMOD correctly localized at the apical membrane, tethered via GPI-anchor (green).
