# Supplementary material for: Disrupted uromodulin trafficking is rescued by targeting TMED cargo receptors
Source: J Clin Invest. 2024 Dec 16;134(24):e180347. doi: 10.1172/JCI180347 (PMC11645142; doi:10.1172/JCI180347)

Figure 2. A. Uncropped Western Blots.

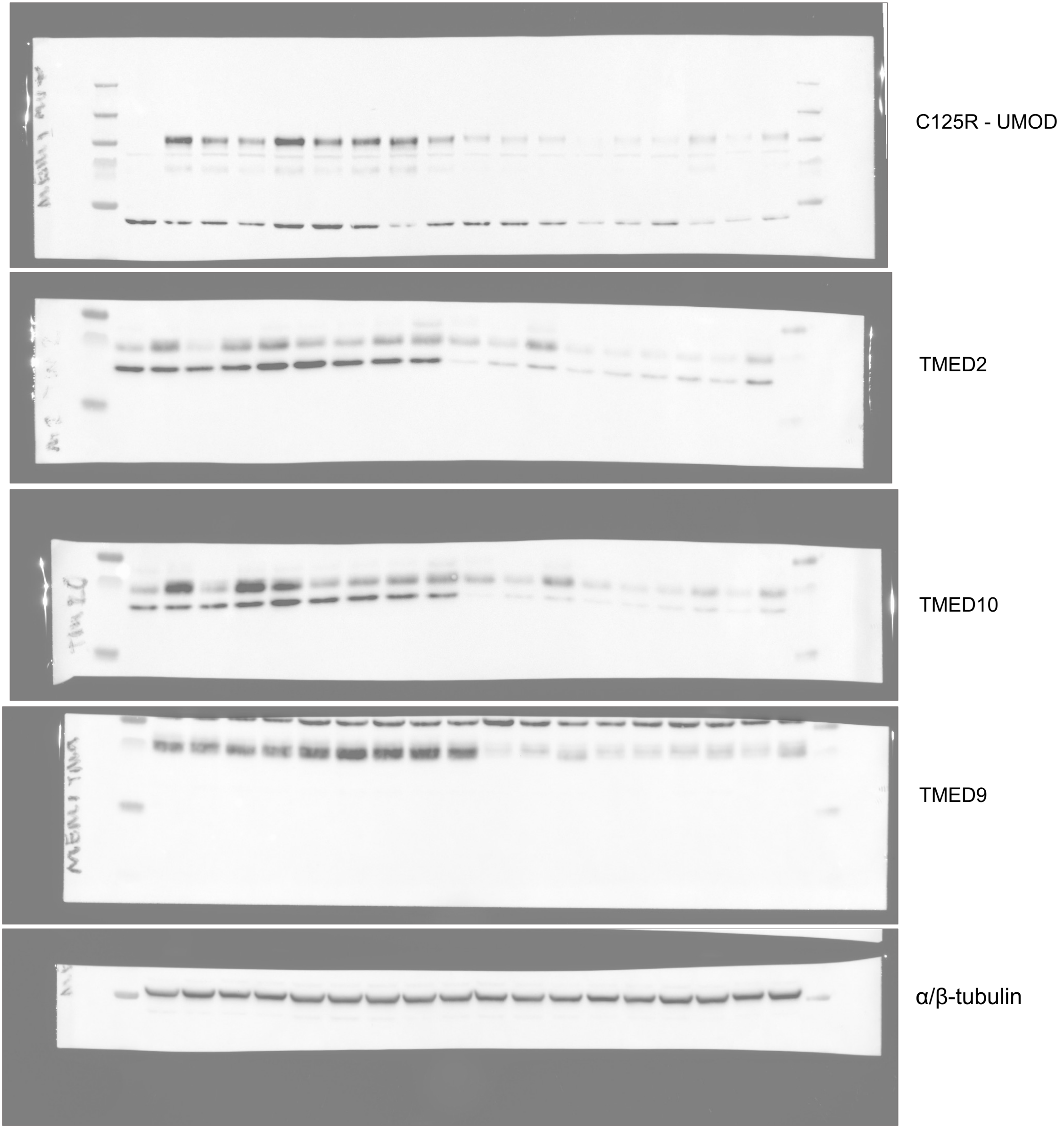

Figure 2. A. For the final quantification, we ran more mice with an additional loading control: Vinculin

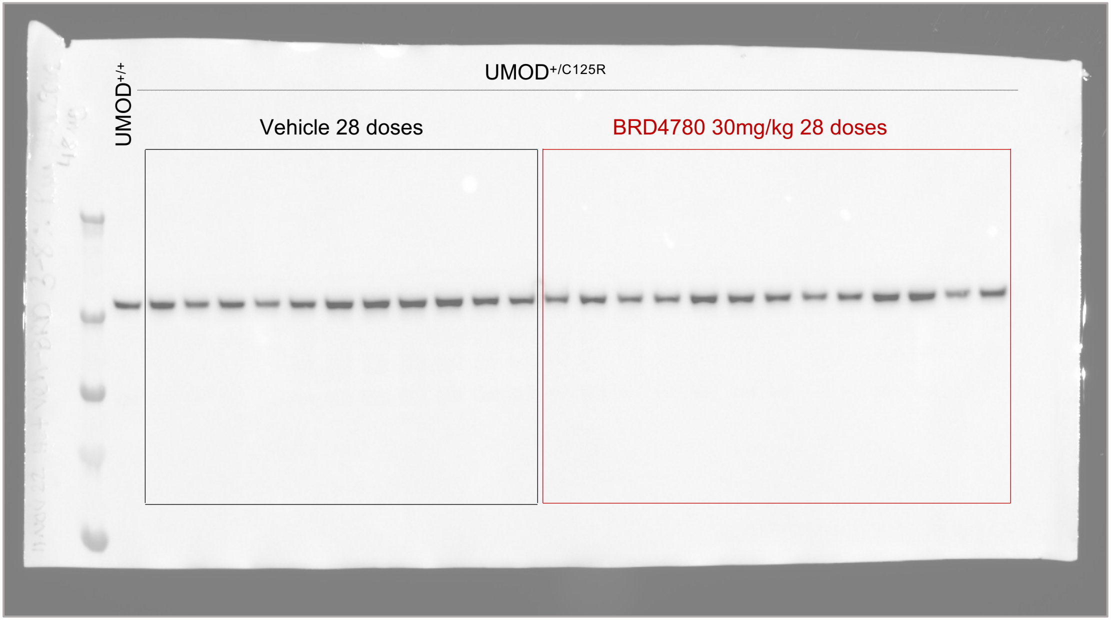

Vinculin-HRP
(CST 1:1000)

Figure 3. C. Uncropped Western Blots. Highlighted in red boxes are the explanations for the extra lanes, not shown in the cropped version.

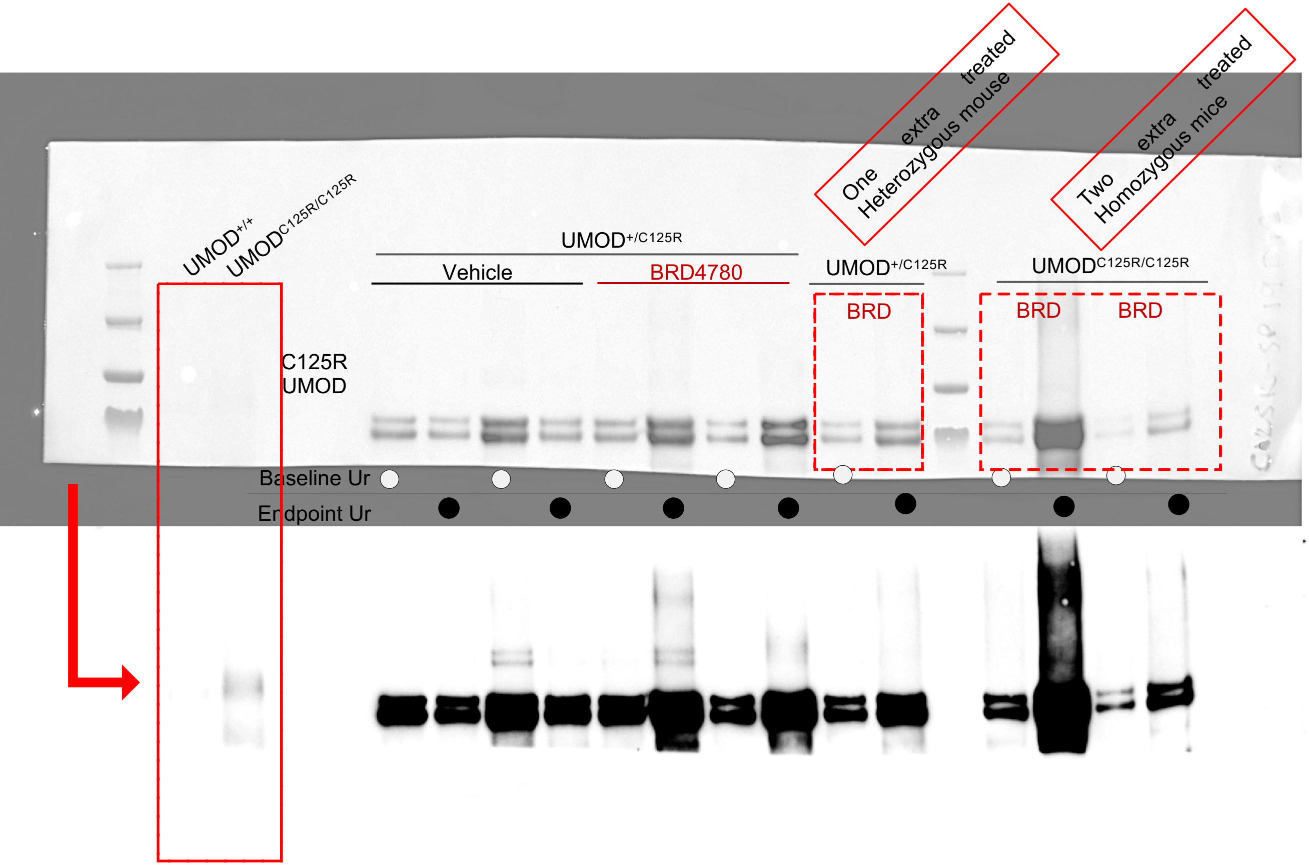

To be able to see the left part of the blot (where we ran the whole-kidney lysates) we had to use a different exposure to what is shown in the upper panel.

Figure 5. A. Uncropped Western Blots.

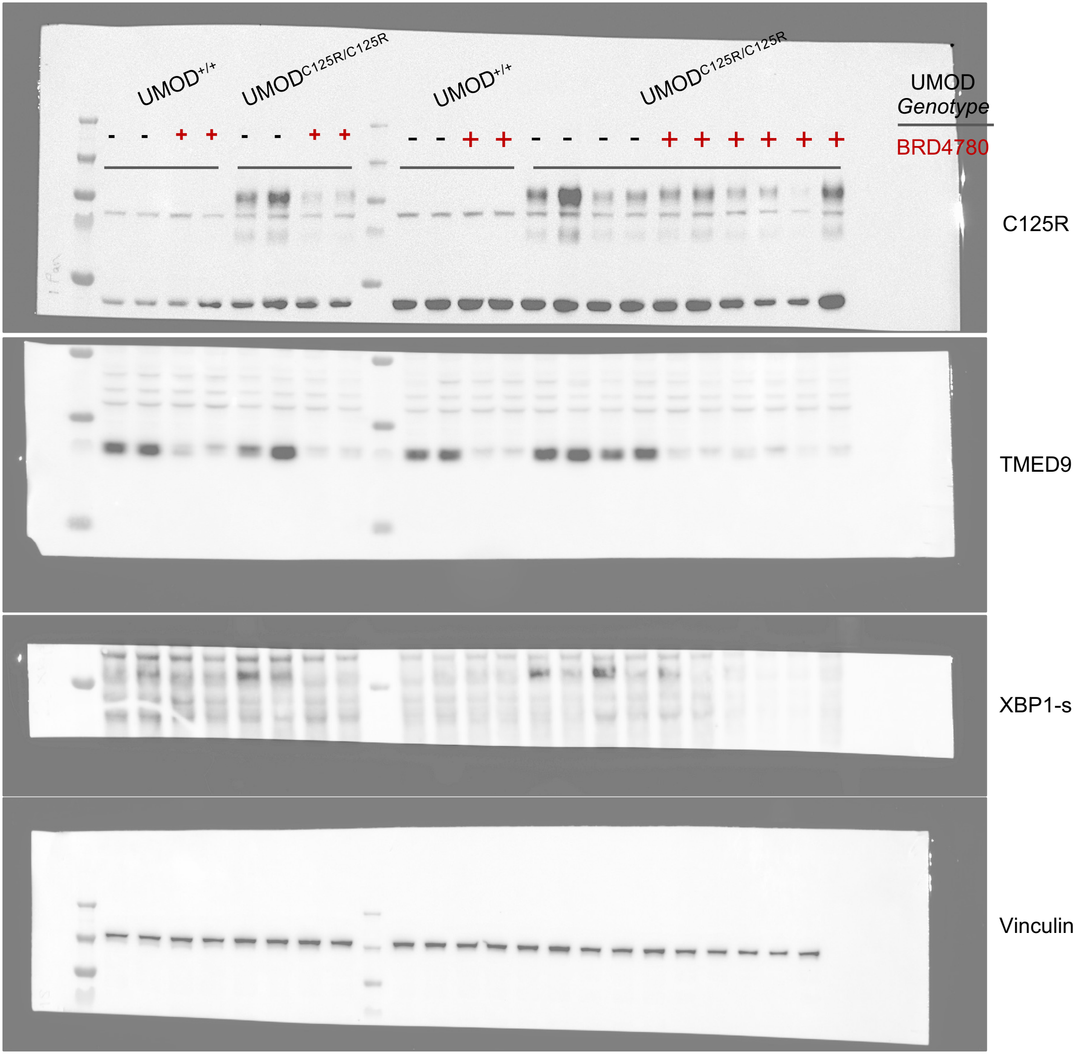

Figure 5. B. Uncropped Western Blots.

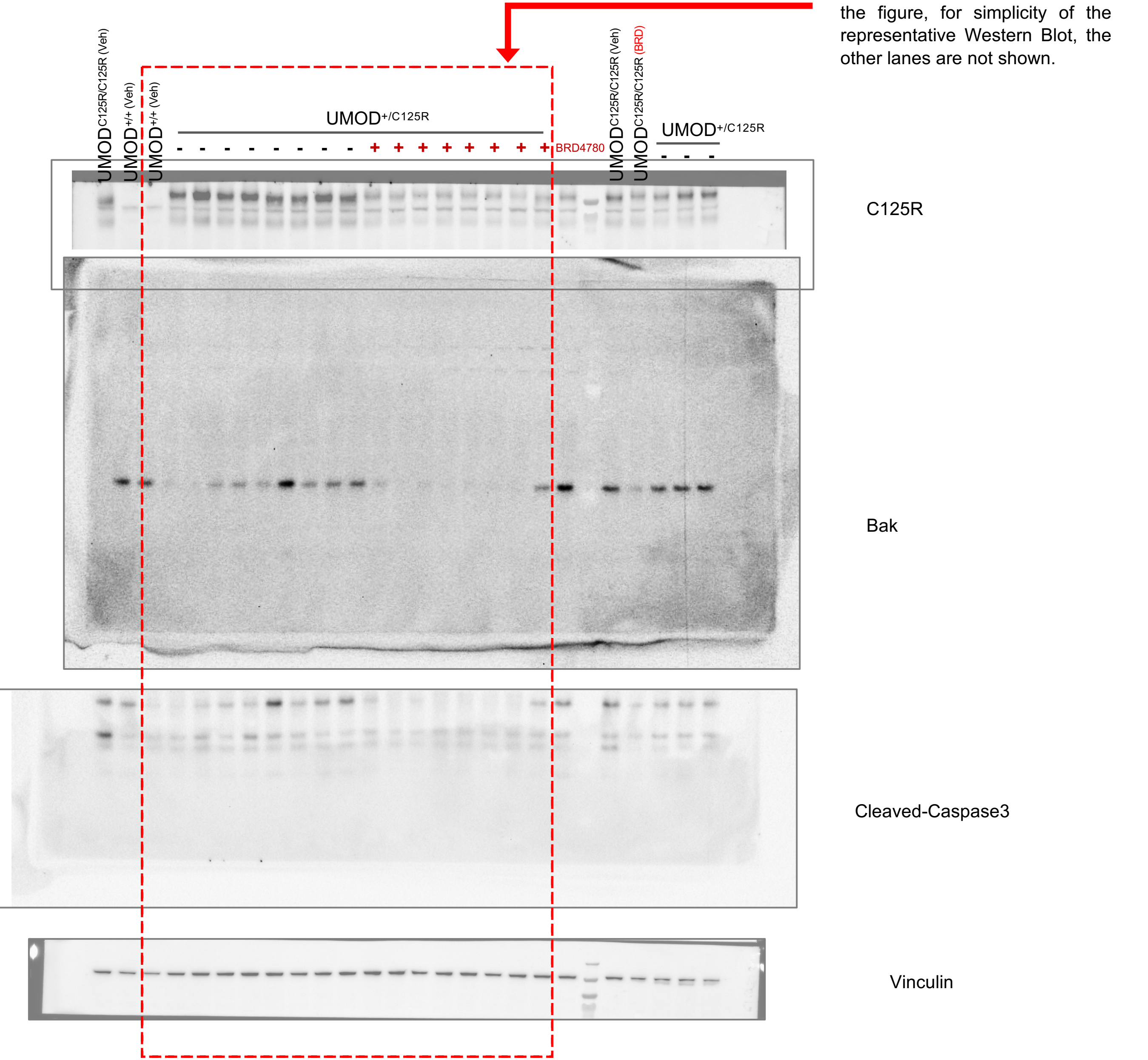

Supplement: Unedited blot and gel images [file jci-134-180347-s009.pdf]
